# Revenue vs expenditure based fiscal consolidation: the pass-through from federal cuts to local taxes

**DOI:** 10.1007/s10797-021-09682-1

**Published:** 2021-09-02

**Authors:** Luigi Marattin, Tommaso Nannicini, Francesco Porcelli

**Affiliations:** 1 University of Bologna, Bologna, Italy; 2 IGIER, Bocconi University, Milan, Italy; 3 University of Bari, Bari, Italy

**Keywords:** Fiscal consolidation, Intergovernmental grants, Difference-in-discontinuities, H2, H77, H87, D7

## Abstract

A growing literature emphasizes that the output effect of fiscal consolidation hinges on its composition, as the choice of increasing revenues vs cutting expenditure is not neutral. Existing studies, however, underscore the role of local governments in a federal setting. Indeed, transfer cuts at the central level might translate into higher local taxes, changing the effective composition of the fiscal adjustment. We evaluate this transmission mechanism in Italy, where municipalities below the threshold of 5,000 inhabitants were exempted from (large) transfer cuts in 2012. This allows us to implement a difference-in-discontinuities design in order to estimate the causal impact of transfer cuts on the composition of fiscal adjustment, also because tight fiscal rules impose a balanced budget on Italian municipalities. We find a pass-through mechanism by which local governments react to the contraction of intergovernmental grants by mainly increasing taxes rather than reducing spending. From a political economy perspective, this revenue based fiscal consolidation is driven by local governments with low electoral competition and low party fragmentation.

## Introduction

Recent works in both macroeconomics and political economics show that the output effect of fiscal consolidation depends on its composition, as the choice of increasing revenues vs cutting expenditure is not neutral from both an economic and a political perspective (see Berndt et al. 2012; Alesina and Ardagna 2013, Alesina et al. 2017, 2019).^1^ In particular, the evidence summarized by Alesina et al. (2019) shows that expenditure based fiscal consolidations, on average, have a smaller contractionary effect than tax based fiscal consolidations. Yet, existing studies underscore the role of local governments in a federal setting. As transfer cuts at the central level might translate into higher local taxes, changing the effective composition of the national fiscal adjustment, any macro evaluation of fiscal consolidations should take this transmission mechanism into account.

What also motivates us is the possibility of exploring the presence of local governments’ asymmetric responses to variation in transfers. In other words, the flypaper effect that we usually observe when the flow of transfers goes up, inducing overexpansion of local expenditure, might be absent in the opposite case of transfer cuts. In this case, local governments might use their tax autonomy to compensate for the reduction in transfers, just reshaping revenues’ composition to avoid spending cuts.^2^

We isolate this mechanism in Italy, where we can causally evaluate the fiscal policy reaction of local governments to a large fiscal consolidation effort imposed at the central level. By doing so, we also join a growing literature that uses variation across cities or regions to identify the impact of economic shocks of interest to macroeconomists, as causal inference is usually difficult with the use of cross-country variation alone.^3^

Italy is the ideal testing ground for the causal evaluation of policy responses to the need of fiscal consolidation. Like many other Western economies, Italy went through a sharp fiscal consolidation in the aftermath of the Great Recession, resulting in a public deficit reduction from 5.3% of the GDP in 2009 to 2.1% in 2018. In the period 2010–2015, approximately one third of this fiscal consolidation occurred through a permanent cut in federal transfers to municipal governments, which were reduced by 8.6 billions of euros (out of a 25.1-billion deficit reduction in nominal terms). As the current public spending of Italian municipalities amounted to 39.6 billions in 2010 (excluding expenditure for local public transport and waste management), this transfer reduction had a sizable impact on the local governments’ fiscal position, and forced them to either increase local taxes or reduce spending, also because a tight balanced-budget constraint is in place and no deficit is allowed at the local level. This environment—which is common to many other countries with a certain degree of decentralization—allows us to investigate the transmission mechanism from a reduction in federal transfers to the composition of fiscal adjustment at lower layers of government. Moreover, as municipalities below the threshold of 5000 inhabitants were exempted from most of the transfer cuts, we can implement a difference-in-discontinuities design to causally evaluate the quantity and quality of the fiscal adjustment effort. Our identification strategy also benefits from the high level of Italian municipalities fragmentation: considering that 75% of them has less than 5000 inhabitants, even a narrow window around this population threshold provides a good sample size.

In particular, local governments in municipalities below the threshold of 5000 inhabitants were exempted from (large) transfer cuts in 2012. This allows us to implement a difference-in-discontinuities design (see Grembi et al. 2016; Eggers et al. 2018) in order to estimate the causal impact of transfer cuts on the composition of fiscal adjustment, by controlling at the same time for confounding policies at 5000 and for time shocks in 2012. In fact, the 5000 threshold is also used in Italy to define the strictness of fiscal rules and the wage paid to local mayors (which has been shown to affect both their quality and performance), but neither of these two policies changed in 2012.^4^ Of course, the year 2012 also affected the Italian economy and public finance because of other factors, but all of them were common to municipalities just below and just above 5000 inhabitants. Therefore, as we formally show in our econometric framework, the combination of the time variation before/after 2012 and the discontinuous variation at 5000 allows us to identify a causal effect.^5^

Our empirical findings disclose a pass-through mechanism by which local governments react to the contraction of intergovernmental grants by mainly increasing taxes rather than reducing spending. In particular, real estate taxation at the local level is suddenly increased in municipalities that have enough fiscal space to do that. From a political economy perspective, we find that this revenue based fiscal consolidation is mainly driven by municipalities with low electoral competition and no party fragmentation in the government coalition. As tax hikes are faster to adopt and bring revenues more rapidly than expenditure cuts, the government may be prone to adopt them to realize the fiscal adjustment, unless it faces (external or internal) political competition by politicians that have an incentive to emphasize the tax increase in the public discussion and campaign against the government.

Our study contributes to different strands of the literature in both macroeconomics and political economics. Moreover, we also provide a small contribution to the growing literature, recently surveyed by Agrawal et al. (2021), on how local governments set policy in a multi-level environment, interacting with other jurisdictions and the central government’s policy goals. In particular, we focus our attention on a less-studied form of interaction, investigating how local governments’ behaviour may alter central government’s fiscal policy crowding-out federal spending cuts increasing local taxes.

The analysis of fiscal adjustments has mainly been conducted at the national level, mostly in the attempt to quantify the output effects of revenue based as opposed to expenditure based fiscal consolidation plans (see Giavazzi and Pagano 1990; Alesina et al. 1998; Forni et al. 2010; Alesina and Ardagna 2013, Erceg and Lendè 2013, Yang et al. 2015, Alesina et al. 2019). At the sub-national level, aside from the vast literature on political budget cycles (see Brender 2003; Brender and Drazen 2005; Drazen and Eslava 2010), the link between changes in transfers from upper-tier governments and the fiscal policy reaction by lower-tier governments has not been widely explored.^6^

The literature on sub-national fiscal policy can broadly be divided into two categories: the first looks at the determinants of the distribution of transfers from the central government to local governments; the second investigates their effects on fiscal policy decisions at the local level. In the first strand, attention has mainly been devoted to verify how much the territorial distribution of transfers is affected by political considerations.^7^ The second strand is the closest to our contribution. Central governments can affect local fiscal policy behavior in two ways: by changing the amount of grants or by imposing non-monetary restrictions such as tax and expenditure limitations. In this respect, Skidmore (1999) looks at local tax limitations imposed in US states between 1976 and 1990, and finds that they produced a reduction in constrained revenue sources but a parallel increase in unconstrained ones. In other words, they favored a reshuffling of the local budget on the revenue side, leaving pretty much unaltered the spending level.

The most direct way to affect local governments’ fiscal behavior is undoubtedly by changing the level of transfers. In this regard, the literature has mainly focused on the so-called “fly paper effect,” namely the overreaction of local expenditure to changes in transfers from upper-tier governments, as opposed to the reaction of local spending to changes in local income (see, among others, Hines and Thaler 1995; Bailey and Connolly 1998, and for surveys see Hamilton 1983 and Inman 2008). Some empirical evidence of flypaper effects has been found in the US (Case et al. 1993; Knight 2002, and more recently Leduc and Wilson 2017), UK (Gemmel et al. 2002), Norway (Tovmo and Falch 2002), Sweden (Dahlberg et al. 2008), Gernmany (Baskaran 2016) and Braliz (Cruz and Silva 2020). More recently, Becker et al. (2020) find evidence in support of the flypaper effect through laboratory experiments. Although these studies provide causal estimates, they do not find evidence of asymmetric behaviour, that is a less than proportional reduction of local public spending when central grants are reduced (which is specifically the scope of our paper). As for the Italian case, Levaggi and Zanola (2003) and Legrenzi (2009) both find descriptive evidence of downward inflexibility of, respectively, regional and municipal public spending in the event of transfer reduction. Gennari and Messina (2014) do not find any robust result in terms of fiscal replacement, that is, municipalities reacting to smaller transfers by increasing their own tax revenues. Our paper stems from these mixed results and implements a quasi-experimental design to estimate the causal effect of transfer cuts on both local taxes and spending, as well as heterogeneous responses by the underlying political environment.^8^

The rest of the paper is organized as follows. Section 2 sketches a simple theoretical framework that we use to interpret the empirical results. Section 3 describes the institutional setting and the data sources. Section 4 presents the econometric framework. Section 5 discusses the main findings and robustness checks. Section 6 concludes.

**Table 1:** Main determinants of total transfer cuts, OLS estimates on standardized and euro per capita variables

	Standardized variables			Variables in euros per capita		
Population $> 5000$ inhab.	0.543***		0.534***	36.20***		35.57***
Fiscal capacity (property tax)		0.380***	0.417***		0.0708***	0.0778***
Cadastral values		0.232***	0.0985***		0.0271***	0.0115***
R-squared	0.534	0.373	0.909	0.534	0.373	0.909

Dependent variable $=$ Total transfer cuts, OLS point estimates on municipalities between 1,000 and 10,000 inhabitants, year 2012. Total observations 3,457.

Robust standard errors are in parentheses. Significance at the 10% level is represented by *, at the 5% level by **, and at the 1% level by ***

**Table 2:** Descriptive statistics of outcome variables

Variable		All sample		Treatment group (pop > 5000)		Control group (pop < 5000)	
		Mean	Std. Dev.	Mean	Std. Dev.	Mean	Std. Dev.
Treatment	Dummy	0.2669	0.4424	1	0	0	0
Post treatment	Dummy	0.0677	0.2512	0.2536	0.4351	0	0
Transfer cuts (total)$^{(1)}$	(Euro per capita)	42.41	22.95	68.70	16.11	32.46	16.23
Transfer cuts (dl 112/2008)$^{(1)}$	(Euro per capita)	3.29	1.22	2.61	0.86	3.53	1.25
Transfer cuts (dl 78/2010)$^{(1)}$	(Euro per capita)	10.18	17.55	37.94	9.37	0	0
Transfer cuts (dl 201/2011)$^{(1)}$	(Euro per capita)	21.68	13.18	21.71	12.04	21.68	13.57
Resident population	n.	3764.7	2348.3	7108.9	1399.4	2547.1	1120.3
Current expenditure	(Euro per capita)	588.45	213.19	520.51	166.96	612.38	222.37
Capital expenditure	(Euro per capita)	309.04	476.00	182.64	220.15	353.56	530.76
Property tax	(Euro per capita)	152.80	102.92	158.03	94.630	150.95	105.62
Local income tax	(Euro per capita)	46.054	23.404	51.435	23.944	44.159	22.913
Fees (net of waste manag.)	(Euro per capita)	65.545	72.446	54.572	50.228	69.410	78.431
Total tax revenues	(Euro per capita)	264.40	144.77	264.04	127.66	264.52	150.34
Prop. tax rate (main dwellings)	(Per thousand)	5.0471	0.8406	4.9677	0.7949	5.0746	0.8542
Prop. tax rate (sec. properties)	(Per thousand)	6.8413	1.2475	7.0250	1.3198	6.7770	1.2147
Local income tax	(Per thousand)	0.4012	0.2391	0.3968	0.2639	0.4027	0.2296
Progressive local income tax	Dummy	0.1539	0.3609	0.2291	0.4203	0.1275	0.3335
Obs.	n.	13795		3682		10113	

Average values computed for year 2012 at the end of the sample period

**Fig. 1:**
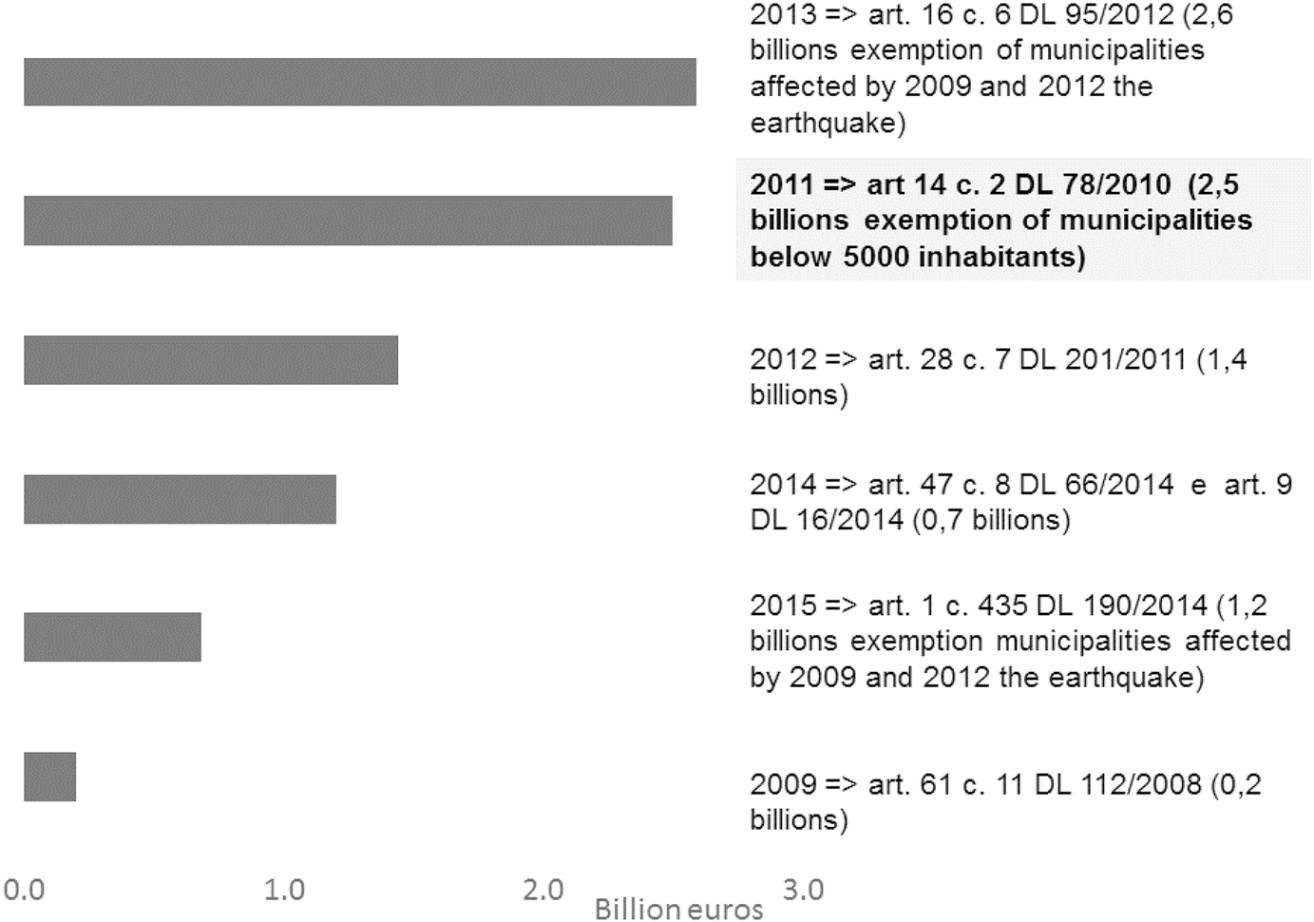
Transfer cuts (*source: Ministry of Internal Affairs*)

**Fig. 2:**
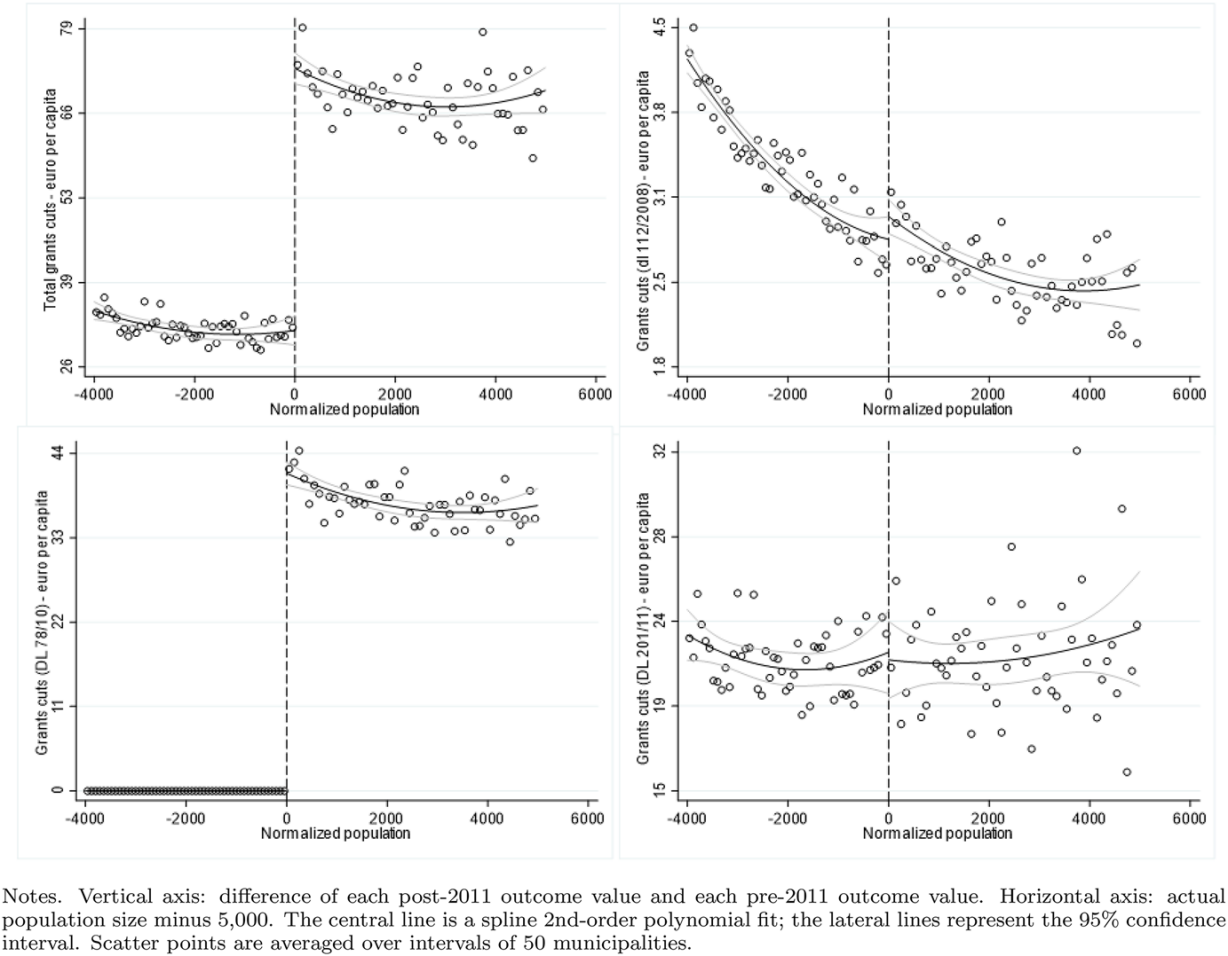
Difference-in-discontinuities, transfer cuts

**Fig. 3:**
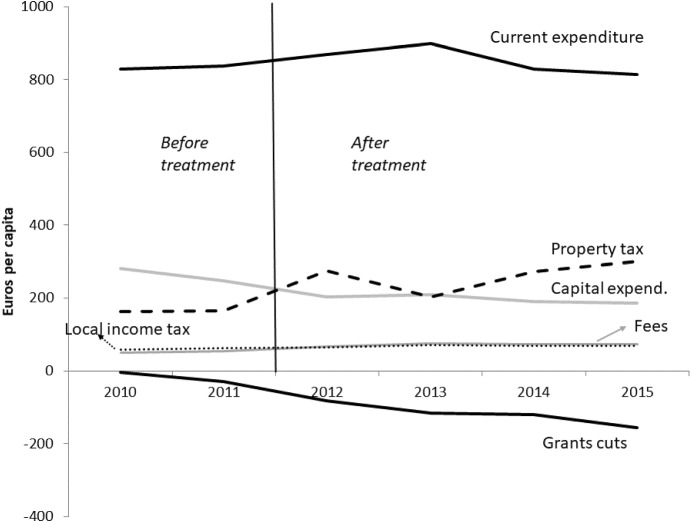
All municipalities, main financial variables (*source: Ministry of Internal Affairs*)

**Fig. 4:**
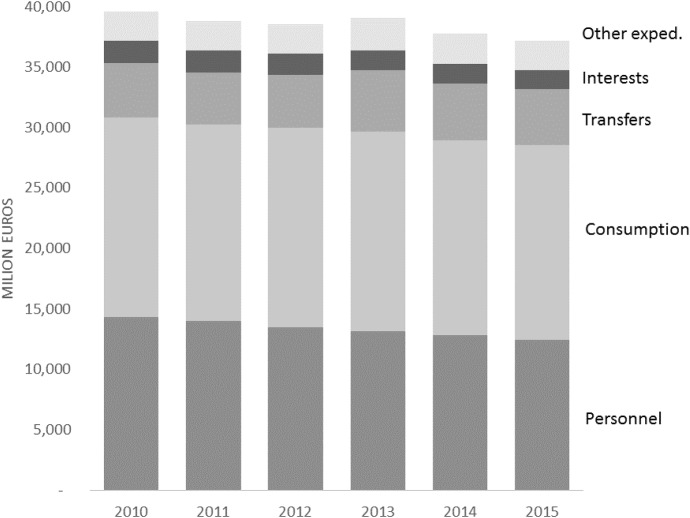
All municipalities, current expenditure composition (*source: Ministry of Internal Affairs*)

## Theoretical preliminaries

The purpose of this section is to set the stage for the empirical analysis by providing a simple theoretical framework, in order to depict the relevant relations taking place in an economy where the central government reduces grants in favor of local governments. The private component of aggregate demand is approximated by a representative agent deriving utility from private consumption and both central and local public spending (as in Barro 1990; Turnovsky and Fisher 1995; Gong and Zou 2002). We adopt a standard two-tier system—as in Gong and Zou (2002)—with a central and a local government.

Based on the scope of our analysis, we assume that while the central government is fully benevolent, the local government has an utility-enhancing component based on the amount of local public spending. The former levies a uniform income tax at a flat rate of $\tau ^Y$, whereas the latter a property or capital tax $\tau ^K$.^9^ Central and local government spending are denoted, respectively, by *G* and *g*. Furthermore, central government transfers in favor of the local government are indicated by $\Gamma$. Both government tiers are subject to a balanced budget constraint.

Agents play a Stackelberg game with the following timing: the central government chooses its policy tools (*G*, $\tau ^Y$, $\Gamma$), the local government does the same (*g*,$\tau ^K$), and then the representative agent chooses its optimal consumption and saving plan.^10^

The representative agent’s discounted utility (twice differentiable and under Inada conditions) is given by:

1 $$\begin{aligned} U_t = \sum _{t=0}^{\infty }\beta ^t u (C_t, G_t, g_t) \end{aligned}$$

with $0<\beta <1$ being the exogenous discount factor and $C_t$ private consumption. The local government’s welfare function has an additive component *W*(*g*), with $W'(g)>0$, capturing the political economy effect of a larger budget. In other words, local governments also gain utility from local public spending, because of either politicians’ rent-seeking or some clientelistic exchange with interest groups at the local level.

The representative agent chooses consumption and next-period capital stock in order to maximize (1) under usual budget constraint; local (central) government chooses local (central) public spending and local (central) tax instrument so to maximize welfare function (1) under balanced budget constraints. Consequently, the three optimality conditions are:

2 $$\begin{aligned} U'(C_t) = \beta U'(C_{t+1}) \left[ (1--\tau _{t+1}^Y) \frac{\Delta Y_{t+1}}{\Delta K_{t+1}} + (1--\tau _{t+1}^{K})\right] \end{aligned}$$

3 $$\begin{aligned} U'(C_{t+1})\lambda _{2,t} = [U'(g_{t+1})+W'(g_{t+1})]K_{t+1} \end{aligned}$$

4 $$\begin{aligned} \lambda _{2,t}U'(C_{t+1}) \frac{\Delta Y_{t+1}}{\Delta K_{t+1}}= U'(G_{t+1}) \end{aligned}$$

Where $U'(C)$ is the marginal utility of private consumption and $\lambda _{2,t}$ is the utility price of an additional unit of private consumption. The equilibrium is pictured by a vector of endogenous variables [$K_{t+1}$, $C_t$, $G_t$, $g_t$, $\tau _{t+1}^{K}$, $\tau _{t+1}^{Y}$, $\Gamma _t$] satisfying the system composed by (2)–(4) and the agents’budget constraints.

Solving the system in steady state and assuming that $U(G)=\log (G)$ and $U(g)=\log (g)$ and $W(g) = log(g)$, we get to the following:

5 $$\begin{aligned} C = \frac{1-\tau ^Y}{\tau ^Y}(G+g)-K\left( \frac{\tau ^K}{\tau ^Y}\right) \end{aligned}$$

6 $$\begin{aligned} \frac{1}{\beta } = \tau ^Y - \frac{\tau ^K}{\tau ^Y} \end{aligned}$$

7 $$\begin{aligned} g = - \tau ^K YK + \frac{\tau ^K}{\tau ^Y} \frac{K}{2} \Gamma \end{aligned}$$

where the absence of a time subscript denotes the value of a variable in a non-stochastic steady-state. Our simple theoretical framework was designed to understand what happens when the central government reduces transfers to the local government. From Eq. (7) we can derive the two steady-state relations that serve our purpose:

8 $$\begin{aligned} \frac{\Delta g}{\Delta \Gamma } = \frac{\tau ^K}{\tau ^Y} \frac{K}{2} > 0 \end{aligned}$$

9 $$\begin{aligned} \frac{\Delta \tau ^K}{\Delta \Gamma } = - \frac{2g \tau ^Y K}{(K \Gamma -\tau ^{Y} KY)^2} < 0 \end{aligned}$$

What happens when the central government cuts transfers? We can isolate three empirical predictions.

i The local government—see Eq. (8)—reduces local public spending. The size of this reduction is bigger the higher the steady-state local tax rate (because the fiscal effort is already strong) and the lower the income tax rate.
ii The local government—see Eq. (9)—increases the local tax rate. The size of this increase is bigger the higher the steady-state level of local public spending (this is probably due to the functional form chosen for *U*(*g*)) and the income tax rate (this is consistent with the previous result). The derivative of (9) with respect to the steady-state capital stock reads:
  10 $$\begin{aligned} \frac{\Delta \left( \frac{\Delta \tau ^K}{\Delta \Gamma } \right) }{\Delta K} = \frac{g \tau ^Y (\Gamma - \tau ^Y)(K-2)}{\Delta [K(\Gamma -\tau ^{Y} Y]^3} \end{aligned}$$
  The sign of Eq. (10) is the same as $(K--2)$. Therefore, if the endowment of capital is high, the increase in the local capital tax rate (following a reduction in federal transfers) is higher, in order to exploit the wider tax base.^11^
iii The increase in the local tax rate following a grant reduction is higher the higher the utility $W(g)=log(g)$ accruing from the political game at the local level:
  11 $$\begin{aligned} \frac{\Delta \left( \frac{\Delta \tau ^K}{\Delta \Gamma } \right) }{\Delta g} = \frac{-2 \tau ^Y K }{\left( K \Gamma - \tau ^Y K Y \right) ^2} \end{aligned}$$
  as $W'(g) = \frac{1}{g}$, it is clear that the higher the marginal utility stemming from the use of local public spending, the higher the size of the pass-through from federal cuts to local taxes.

Although a direct prediction of asymmetric flypaper response goes beyond the scope of our theoretical analysis and is left to further research, we contribute to the existing literature providing an analytical explanation to three possible channels that can increase the possibility of fiscal replacement (i.e. an increase in local taxes rather than a reduction in local expenditure in response to transfers cuts): (1) low fiscal effort ; (2) high tax base ; (3) high marginal utility of expenditure.

It is interesting to notice that most of the studies in the literature (Volden 1999; Melo 2002; Deller and Maher 2006, 2009; Sour 2013) are in line with our channels. However, they consider the dependency on central governments’ grants (typical of poorer jurisdictions) and the rigidity of local expenditures (stimulated by bureaucratic pressures) the leading causes of fiscal replacement without providing any robust theoretical background. Alternative explanations, instead, consider the policymaker mental accounting approach of treating losses and gains differently (Heyndels and Van Driessche 2002), and the incumbent’s ideology coupled with the propensity to deficits (Lago-Penas 2008).

Moreover, our theoretical framework indicates in what direction we could structure part of the empirical analysis, identifying three primary sources of heterogeneity that can cause a response to the transfers cuts. First, the level of fiscal effort can foster a pass-through from federal cuts to lower local spending when tax rates are already high before the cuts. Second, the local tax base level that, instead, can support a pass-through from transfers cuts to higher local taxes in wealthier municipalities. Finally, the political environment and the electoral salience of local taxes can mitigate the increase of local revenues in response to the reduction in central government’s transfers when the level of political competition and the incumbent mayor’s electoral concerns are high. In Sect. 5.2, we bring these predictions to the data identifying five sources of heterogeneous response in the fiscal adjustment: (1) the level of municipal fiscal effort; (2) the level of fiscal capacity; (3) the incumbents’ mayor margin of victory; (4) the political fragmentation of the ruling majority; (5) the possibility of the incumbent mayor to compete for another electoral mandate.

**Table 3:** Descriptive statistics of control variables

Variable		All sample		Treatment group (pop > 5000)		Control group (pop < 5000)	
		Mean	Std. Dev.	Mean	Std. Dev.	Mean	Std. Dev.
Cadastral values	(Euro per estate)	440.21	257.60	508.64	266.29	416.31	250.14
Main dwellings	(Per cent)	63.291	11.029	66.234	9.5165	62.263	11.332
Fiscal capacity (property tax)	(Euro per capita)	244.01	156.82	234.25	134.20	247.50	164.03
Fiscal capacity (income tax)	(Euro per capita)	43.411	12.268	45.090	12.388	42.810	12.169
Domestic stability pact (target)	(Euro per capita)	3.0675	28.318	11.634	54.521	0.0601	2.3130
Population (0-2)	(% Total pop.)	2.6822	0.6460	2.8952	0.5304	2.6046	0.6665
Population (3-14)	(% Total pop.)	10.882	1.7261	11.518	1.5063	10.651	1.7429
Population (15-65)	(% Total pop.)	66.204	2.8612	67.127	2.2475	65.868	2.9841
Population (over 65)	(% Total pop.)	21.265	4.5552	19.482	3.7012	21.914	4.6624
Real estate declared income	(Euro per capita)	368.60	218.87	411.02	225.45	353.20	214.37
Declared employment income	(Euro per capita)	6217.8	1881.3	6561.4	1944.3	6093.1	1842.2
Residential estate market values	(Euro per sq meter)	815.71	386.04	924.29	496.88	776.46	328.43
Electoral cycle	(Discrete 0-4)	2.6938	1.2916	2.7388	1.3270	2.6776	1.2783
Term limit	Dummy	0.1934	0.3950	0.3751	0.4842	0.1282	0.3343
Electoral year	Dummy	0.2366	0.4250	0.2357	0.4245	0.2368	0.4251
Turnout	(Per cent)	76.315	7.9831	76.637	6.0960	76.200	8.5548
Margin of victory	(Per cent)	17.954	15.289	16.353	13.689	18.546	15.800
Obs.	n.	13795		3682		10113

**Table 4:** The impact of transfer cuts on main outcome variables, difference-in-discontinuity estimates

	DID	DID	LLR	LLR	LPR (2nd order)	LPR (2nd order)
Local income tax	4.994***	4.496***	8.254**	8.371**	9.448	10.91*
*bandwidth*	All sample	All sample	954	954	1000	1000
Fees	2.561**	2.726**	15.74**	14.64**	18.31**	17.87**
*bandwidth*	All sample	All sample	674	674	1000	1000
Property tax	18.00***	11.91***	27.09***	29.53***	24.36	32.81*
*bandwidth*	All sample	All sample	1291	1291	1000	1000
Total tax rev.	25.55***	19.13***	39.76***	45.77***	52.12**	61.60***
*bandwidth*	All sample	All sample	1006	1006	1000	1000
Capital expenditure	42.11***	35.70***	$-$2.985	$-$5.961	37.79	39.11
*bandwidth*	All sample	All sample	1377	1377	1000	1000
Current expenditure	$-$9.607***	$-$8.432***	10.40	10.14	$-$18.03	$-$17.45
*bandwidth*	All sample	All sample	955	955	1000	1000
Control variables	No	Yes	No	Yes	No	Yes

Municipalities between 1000 and 10,000 inhabitants; budget years between 2009 and 2012. The total number of observations corresponds to 13,795.

Difference-in-discontinuity estimates of the impact of grants reductions on fiscal variables in municipalities above 5000 inhabitants after 2011.

Estimation methods: Local Linear Regression (LLR) with optimal bandwidth, as in Eq. (12);

2nd-order spline polynomial approximation (LPR), as in Eq. (13).

All policy outcomes are in euros per capita. Year and municipal fixed effects are included in all specifications

Robust standard errors clustered at the municipality level are in parentheses. Significance at the 10% level is represented by *,

at the 5% level by **, and at the 1% level by ***.

Control variables include: cadastral values, % of main dwellings, fiscal capacity (property tax), fiscal capacity (local income tax), domestic stability pact target, % of population (0-2),

% of population (3-14), % of population (over 65), real estate declared income, declared employment income, residential estate market values, electoral cycle, term limit,

electoral year dummy, municipal election turnout, margin of victory of the incumbent mayor

## Institutional background and data

### Italian sub-national governments

In this section, we provide background information on the Italian system of sub-national governments. This will allow us to show why Italy is a perfect testing ground to estimate the impact of federal cuts on the composition of the fiscal adjustment realized by lower layers of government in a federal system. Italy is a unitary Republic with three layers of sub-national governments. The territory is divided in 20 Regions (five of which have a special statute that gives them higher autonomy from the central government), managed by elected regional governments that account for 19% of total current public expenditure (143 billion euros). The main responsibilities of regional governments are in the following sectors: healthcare; public transportation; complementary social welfare; higher education; and vocational training.

The second layer of the institutional system is represented by 93 Provinces (17 of which are within special regions) and 14 Metropolitan districts (4 of which are within special regions), managed by local administrators appointed among the members of the municipal councils elected within the boundaries of each province or metropolitan district. At this level of government is allocated the 0.8% of total current public expenditure (6 billion euros) in order to provide services related to the maintenance of provincial road network, the management of public high school buildings, environmental protection and, depending on the regions, other delegated functions by regional governments in local public transportation or vocational training.

The third and most important layer of the institutional system is represented by municipalities (*Comuni*), which have a long and important historical tradition in Italy. Municipal governments are ruled by a city council and an executive committee appointed by the elected mayor (*Sindaco*). The council and the mayor are directly elected for a five-year term and are subject to a two-term limit.^12^ As in many other European countries, also in Italy, there is a high level of fragmentation at the municipal level. There exist 7978 municipalities (1351 of which are within special regions); 85% of all municipalities have less than 10,000 inhabitants, 75% less than 5000, 24% less than 1000 inhabitants, while only 6 cities have more than 500,000 inhabitants. At this level of government is allocated 6.8% of total current public expenditure (52.2 billion euros), by which a wide range of essential public services are provided: environment protection and waste management, social services to elderly and disabled persons, childcare and nursery schools, school-related services (such as school meals and transportation), local police, maintenance of municipal roads, management of civil registries, town planning, culture, recreation, and economic development.

In our analysis, we focus on municipalities within normal-statute regions, as they share the same set of fiscal rules. In particular, the current expenditure of these 6627 municipalities is fully financed by local taxes and fees plus horizontal (non earmarked) equalization grants allocated with a system based on historical expenditure up to 2014; after that year a new equalization system based on the difference between standard expenditure needs and fiscal capacity has been gradually introduced with the goal of completely replacing the previous method in 2021. Specific grants are exceptional and earmarked; they are a residual source of funding provided by the central or the regional government, in favor of municipalities with specific investment needs.

Municipalities’ own fiscal revenues come from two main sources: (1) local taxes, among which the most relevant are the Property Tax (called “ICI” until 2011 and “IMU” afterward), the tax on waste disposal (called “TARSU” until 2011 and “TARI” afterward), and the local income tax surcharge; (2) local fees related to road and traffic, libraries, theaters and culture, burial services, and other services such as the occupation of public spaces, public billboards, certificates.

According to the Italian Constitution, all local governments are subject to a balanced-budget constraint and fiscal deficit is allowed only to finance capital expenditure. Moreover, as an additional and fundamental fiscal discipline mechanism, all municipalities (with the exception of those below 5000 inhabitants until 2013), provinces, and metropolitan districts must comply with the rules of the “Domestic Stability Pact” (DSP). The DSP was introduced in Italy, as in other European countries, in 1999 after the European Union adopted its Stability and Growth Pact in 1997. According to the rules of the DSP, local governments have to keep their fiscal gap below a specific target fixed by the central government. Since 1999, the definition of the fiscal gap has changed multiple times.^13^

**Fig. 5:**
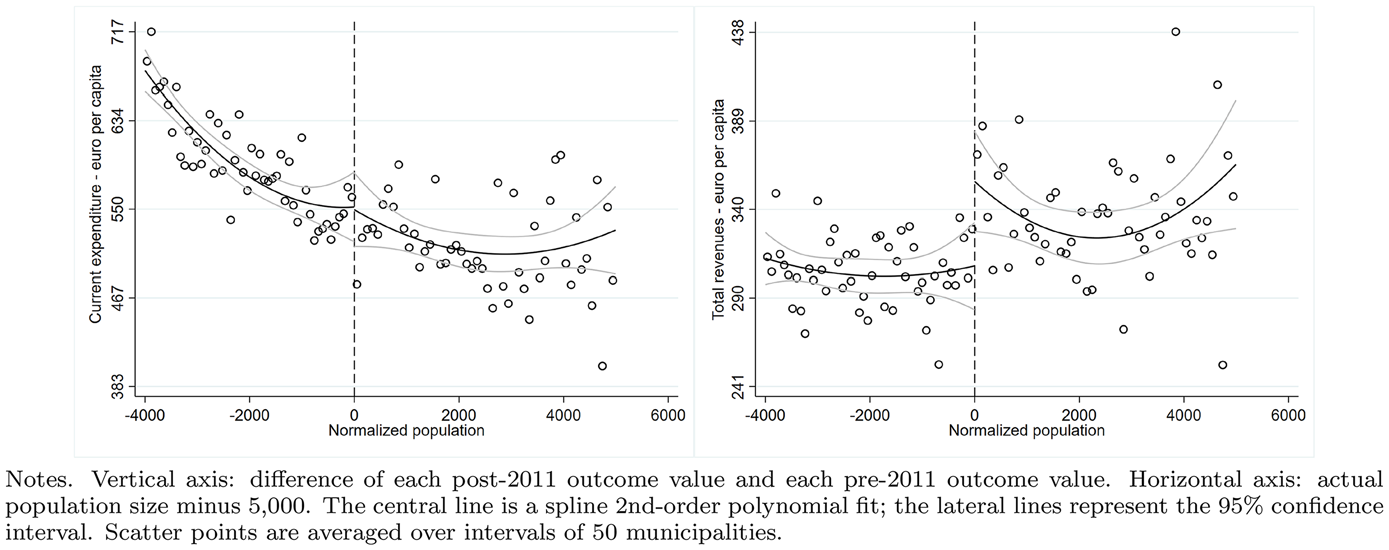
Difference-in-discontinuities, current expenditure and total tax revenues

**Fig. 6:**
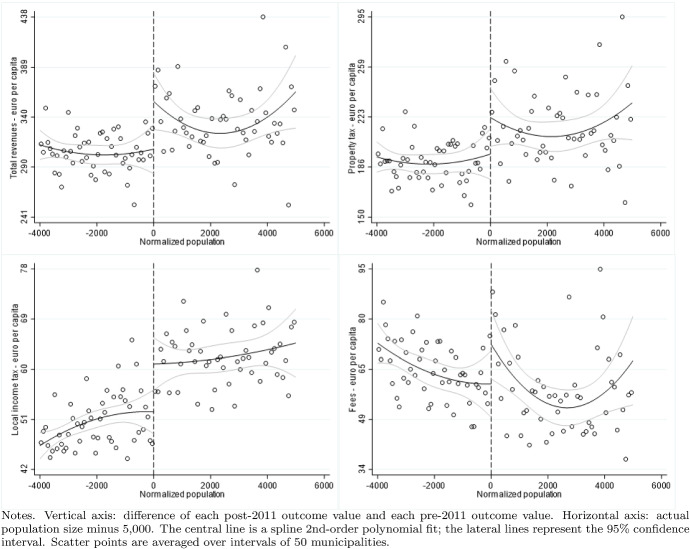
Difference-in-discontinuities, main components of tax revenues

**Fig. 7:**
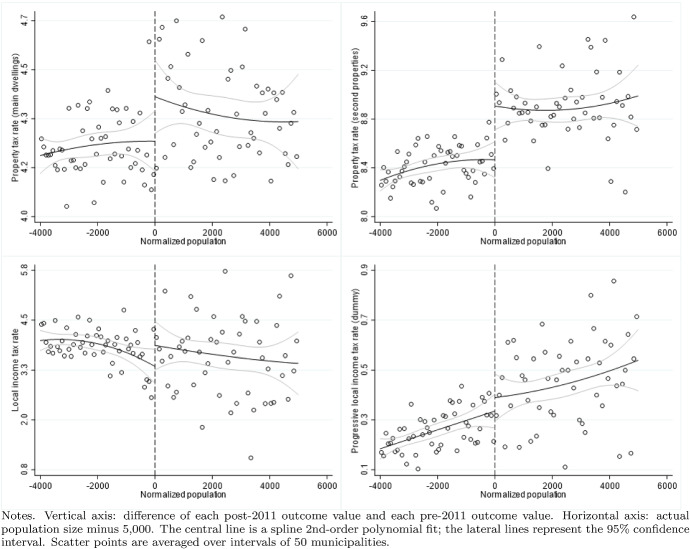
Difference-in-discontinuities, municipal tax rates

### Fiscal consolidation

In the aftermath of the financial crisis, starting in 2010, the Italian government has implemented an intense program of spending cuts.^14^ In the period 2010–2015, approximately one third of the fiscal consolidation occurred through a permanent cut in transfers to municipal governments, which were reduced by 8.6 billion euros, corresponding to roughly 16% of current expenditure or 33% of capital expenditure at the municipal level. As a result of these cuts, in 2015 the vertical component of the equalization grants was abolished, and the equalization system became horizontal.

Figure 1 provides a snapshot of the different laws through which the cuts in federal transfers were gradually imposed. Our empirical analysis exploits the 2.5 billion euros reduction introduced by the art. 14 par. 2 of the decree 78/2010, which became effective in 2012. This reduction in federal transfers is particularly interesting because municipalities below 5000 inhabitants were fully exempted from it. As far as the other transfer cuts are concerned, none of them exhibits the same exemption, only municipalities affected by the 2009 and 2012 earthquakes were exempted by the 2013 and the final 2015 transfer cuts. In particular, to visualize more details about the structure and the determinants of the transfer cuts, Figure 2 reports the graphical analysis of the discontinuities around the threshold of 5000 inhabitants in the total amount of the three transfer cuts included in our data set (decrees 78/2010, 201/2011, 112/2008). A clear discontinuity is present in the transfer cuts introduced by the decree 78/2010. The remaining two transfer cuts show no discontinuity in the resident population level. Therefore, the discontinuity remains visible in the transfer cuts’ total amount. As shown by the regression analysis reported in Table 1, the threshold of 5000 inhabitants explains half of the total variance in the allocation of the transfer cuts. The level of the fiscal capacity and the tax base of the property tax, two exogenous variables out of the control of the municipal governments, explain the rest of the variability.

Note that there are other municipal policies that also jump at 5000 inhabitants: in particular, the wage of the mayor sharply increases above this threshold (see Gagliarducci and Nannicini 2013) and the DSP is not enforced below this threshold (see Grembi et al. 2016). The wage policy, however, is time invariant, while the DSP exemption at 5000 did not vary until 2013 (which is why we restrict our sample to observations before this year).

Figure 3 shows the trends followed by the main municipal financial variables between 2010 and 2015. Each value corresponds to the national average of the variable expressed in per capita terms, considering only municipalities within the normal-statute regions. Although current expenditure exhibits a slight reduction between 2010 and 2015, its downward trend is less steep if compared with the trend of the cumulative grant reductions over the six years.^15^ A stable decrease of capital expenditure is instead clearly visible, although it was mainly the result of the fiscal constraints imposed by the DSP.

From Fig. 3, we observe a clear increase in the level of the property tax, also due to the 2012 reform passed by the central government as one of the main pillars of the fiscal consolidation program implemented to cope with the consequences of the financial crisis. Between 2011 and 2012, total revenues from the property tax passed from 9.8 billion to 23.8 billion euros, thanks to the revaluation of the cadastral values and to the taxation of the owner-occupied dwellings previously exempted in 2008. However, half of the total tax revenues were retained by the central government in a peculiar form of tax-sharing (only municipal revenues are shown in the graph). Finally, not much variation is visible in the local income tax and in the level of fees.

Figure 4 provides an in-depth analysis of the current expenditure composition in the six years between 2010 and 2015. The graph shows that the reduction of current expenditure observed between 2010 and 2015 mainly comes from the reduction of personnel expenditure, achieved by the introduction of specific limitations on the hiring of new staff. From the analysis of the raw data it seems that the process of fiscal consolidation did not produce, at least in the short run, a visible contraction of total municipal current expenditure. Instead, we observe an increase in the property tax.

This general evidence may lead to the conclusion that mayors reacted to the reduction of grants mainly by increasing local taxes, and therefore there was no transmission to the local level of the spending cuts implemented by the central government. Of course, tons of confounding factors may produce this descriptive evidence. To isolate the causal impact of transfer cuts on the composition of the fiscal adjustment at the local level, we exploit the richness of Italian institutions and data, and we implement a “difference-in-discontinuities” design (see Grembi et al. 2016; Eggers et al. 2018) at the 5000 threshold in 2012. As discussed below, the sharp discontinuity at 5000 allows us to control for time-varying confounders (e.g., the property tax reform in 2012), while the time variation before/after 2012 allows us to control for time-invariant confounders which also jump at the 5000 threshold (e.g., the DSP and mayor’s wage). In the remaining parts of this section, we describe our data and formalize the econometric design of our evaluation exercise.

**Table 5:** The impact of transfer cuts on additional outcome variables, difference-in-discontinuity estimates

	DID	DID	LLR	LLR	LPR (2nd order)	LPR (2nd order)
Local income tax rate	$-$0.0354***	$-$0.0260***	0.0479	0.0428	0.124	0.125
*bandwidth*	All sample	All sample	1227	1227	1000	1000
Progressive local income tax	0.131***	0.112***	0.0320	0.0363	$-$0.0484	$-$0.0340
*bandwidth*	All sample	All sample	1333	1333	1000	1000
Property tax rate (main dwellings)	0.347***	0.311***	0.444**	0.485**	0.388	0.437
*bandwidth*	All sample	All sample	962	962	1000	1000
Property tax rate (second properties)	0.319***	0.319***	0.492**	0.481**	0.517	0.517
*bandwidth*	All sample	All sample	1394	1394	1000	1000
Control variables	No	Yes	No	Yes	No	Yes

Municipalities between 1000 and 10,000 inhabitants; budget years between 2009 and 2012. The total number of observations corresponds to 13,795.

Difference-in-discontinuity estimates of the impact of grants reductions on fiscal variables in municipalities above 5000 inhabitants after 2011.

Estimation methods: Local Linear Regression (LLR) with optimal bandwidth, as in Eq. (12);

2nd-order spline polynomial approximation (LPR), as in Eq. (13).

All policy outcomes are in euros per capita. Year and municipal fixed effects are included in all specifications.

Robust standard errors clustered at the municipality level are in parentheses. Significance at the 10% level is represented by *,

at the 5% level by **, and at the 1% level by ***.

Control variables include: cadastral values, % of main dwellings, fiscal capacity (property tax), fiscal capacity (local income tax), domestic stability pact target, % of population (0–2),

% of population (3–14), % of population (over 65), real estate declared income, declared employment income, residential estate market values, electoral cycle, term limit,

electoral year dummy, municipal election turnout, margin of victory of the incumbent mayor

### Data

In order to construct our dataset as homogeneous as possible, we include in the sample only municipalities between 1000 and 10,000 inhabitants, also excluding those in the special regions and those affected by the 2009 and 2012 earthquakes (as they all were subject to a different set of fiscal rules). Moreover, we restrict the time span to the four-year period between 2009 and 2012, as the rules of the DSP are stable over this period (in particular, the exemption threshold for the DSP shifts from 5000 to 1000 in 2013). Table 2 reports the descriptive statistics of the variables included in the dataset for the entire sample and, separately, for the treatment group (municipalities between 5000 and 10,000 inhabitants) and the control group (municipalities between 1,000 and 5000 inhabitants).

The “treatment” dummy takes value one for municipalities above 5000 inhabitants, which represent 27% of all observations. The “post treatment” dummy takes value one for the year 2012 only for municipalities above 5000 inhabitants, that is, 7% of the total observations and 25% of the municipalities above the 5000 population threshold. The source of the financial information included in the dataset is the municipal budget accounts published yearly by the Italian Ministry of Internal Affairs, and the full list of financial variables includes: transfer cuts, current expenses, capital expenses, local tax revenues, fees, grants, and the DSP target. The Italian Ministry of Finance, upon a specific request, provided information on the distribution of the legal tax rates for the property tax^16^ and the local income tax^17^, and information regarding the structure of the tax base of the property tax considering the average cadastral value and the percentage of main dwellings (owner-occupied main residence). We completed the dataset with the usual set of control variables including information related to the structure of the population by age, the level of average municipal income (divided between employment income and real estate income), the level of municipal fiscal capacity (divided between the property tax and the local income tax), the level of real estate market values and, finally, information about political fragmentation, electoral outcomes, electoral cycle, turnout, and ideology of the elected mayor.

Tables 2 and 3, respectively for the outcome variables and the control variables, compare the means of each variable between the treatment and control groups in our sample. Almost all variables show values of similar magnitude in the two groups. There are, however, few exceptions, in addition to the level of the transfer cuts and the average resident population where the difference is obviously expected: capital expenditure, which shows much higher values for municipalities below the 5000 threshold; the adoption of the progressive structure for the local income tax, that is twice higher in municipalities above the 5000 threshold; and the fiscal gap target of the DSP, which, as discussed above, was not applied to municipalities with less than 5000 inhabitants until 2013 (the average below 5000 is not exactly zero since some municipalities crossed the 5000 threshold in some years).

Regarding electoral variables we can also notice the absence of sharp differences between the two groups: both municipalities below and above 5000 exhibit an average turnout, an average margin of victory, and the probability of facing an election that are close to the national mean (respectively, 76%, 18%, 23%); 38% of the mayors in municipalities above 5000 show a term limit compared to 13% in smaller municipalities.

**Table 6:** The impact of transfer cuts on current expenditure after 2012, difference-in-discontinuity estimate

	DID	LLR	LPR (2nd order)
Current expend. (2013)	$-$9.523***	$-$2.367	$-$49.70
*bandwidth*	All sample	932	1000
Current expend. (2014)	$-$2.495	16.77	8.130
*bandwidth*	All sample	961	1000
Current expend. (2015)	$-$1.810	14.98	$-$6.269
*bandwidth*	All sample	985	1000
Current expend. (2016)	1.704	8.491	$-$34.77
*bandwidth*	All sample	997	1000

Municipalities between 1000 and 10,000 inhabitants; budget years between 2009 and 2012. The total.

number of observations corresponds to 13,795.

Difference-in-discontinuity estimates of the impact of grants reductions on pre-treatment covariates in

municipalities above 5000 inhabitants after 2011.

Estimation methods: Local Linear Regression (LLR) with optimal bandwidth, as in Eq. (12);

2nd-order spline polynomial approximation (LPR), as in Eq. (13).

All policy outcomes are in euros per capita. Year and municipal fixed effects and control variables are

included in all specifications. Robust standard errors clustered at the municipality level are in parentheses.

Significance at the 10% level is represented by *, at the 5% level by **, and at the 1% level by ***

**Fig. 8:**
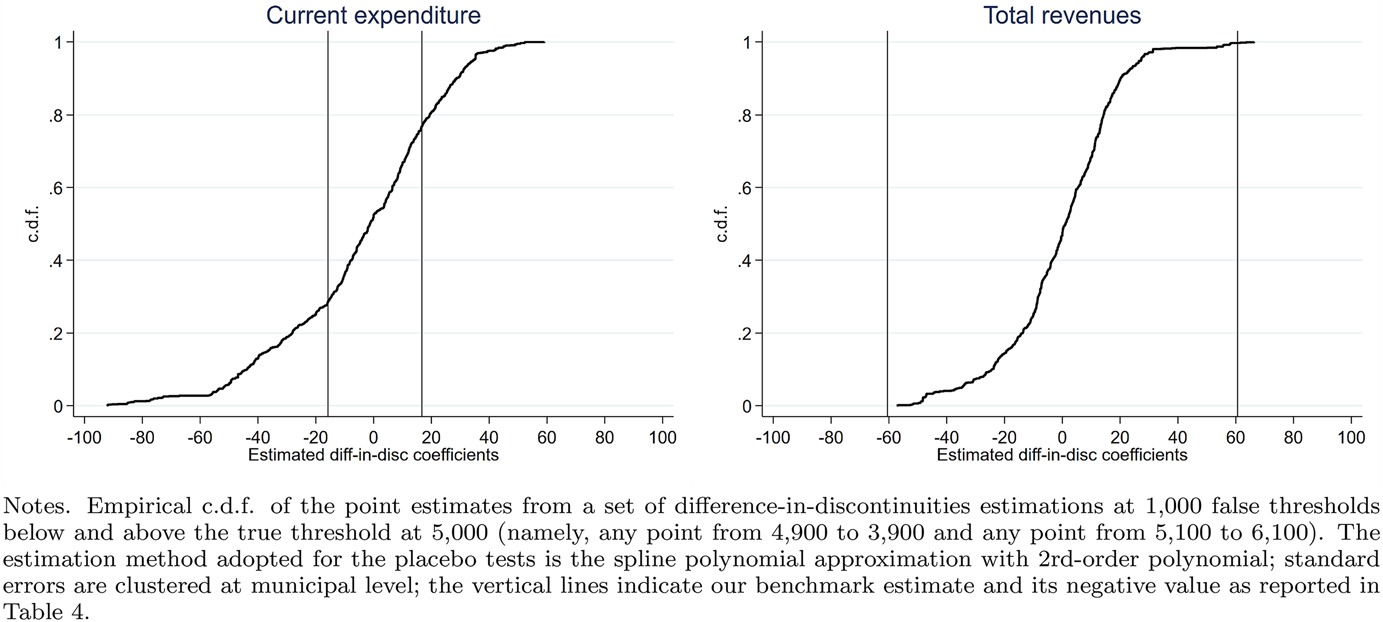
Placebo tests based on permutation methods

**Fig. 9:**
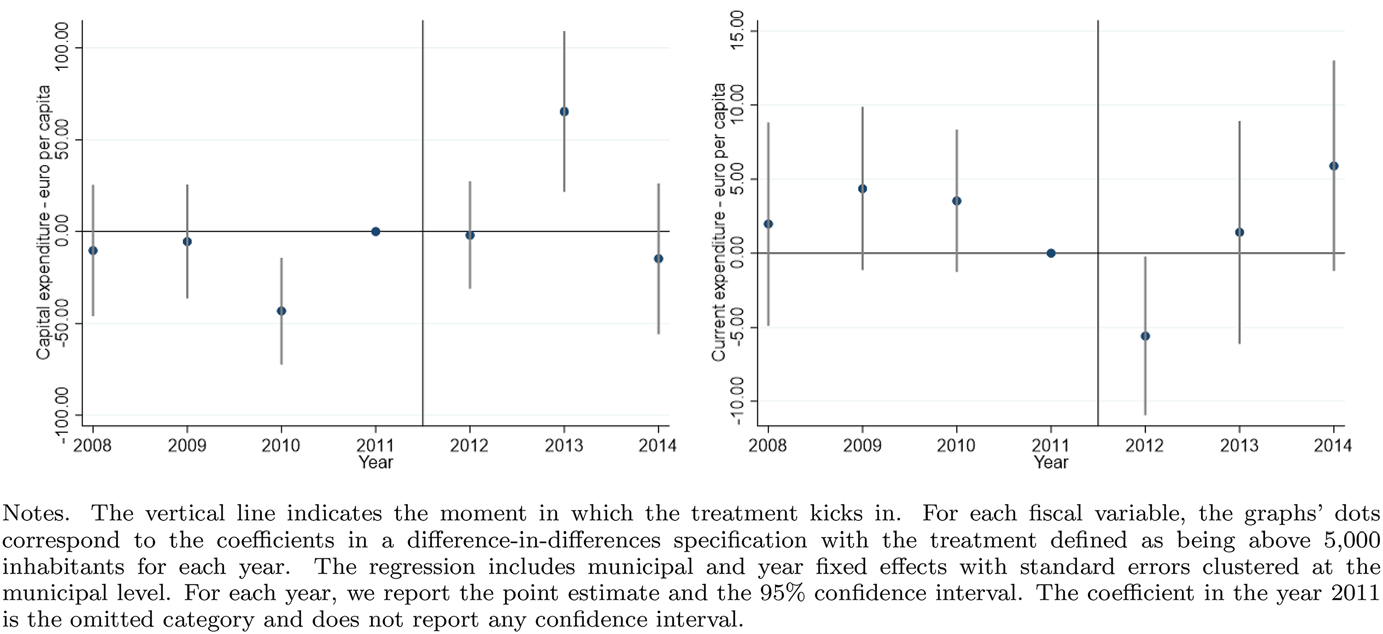
Expenditure variables, pre and post-treatment trends

## Econometric design

In order to identify the causal impact of the 2012 transfer cuts, imposed to municipalities above 5000 inhabitants but not to those below, we need to control for a series of potential confounding factors, which prevent the use of a simple (cross-sectional) regression discontinuity design. The 5000 threshold is in fact associated to a compound treatment, as other municipal policies use the same population cutoff. First, municipalities below 5000 were exempted from the DSP until 2013. Second, the wage of mayors and executive committee members sharply increases at 5000. As discussed above, we restrict our sample to observations before 2013 so that both confounding policies are time-invariant in our analysis. This allows us to control for them by exploiting the time variation before/after 2012. One additional confounding factor is the implementation of the 2012 property tax reform, which increased local tax autonomy. The reform, however, was implemented for all municipalities and this allows us to control for it by exploiting the discontinuity at 5000. In particular, the 2012 cadastral revaluation, which represents the bulk of the reform, consisted of a simple sixty per cent increase of the tax base, uniform across all municipalities and nation-wide. Further specific revaluations of cadastral values are national decisions out of mayors’ exclusive decision power. However, to control any influence produced by the change in the tax base, we have included the average cadastral value computed at municipal level among the control variables. Moreover, another potential confounding source would come from the distribution of the share of owner-occupied main residence across municipalities since they entered the tax base composition in 2012. Although we did not have any discontinuity evidence at 5000 in the percentage of the owner-occupied main residence, we have included this variable in the controls set. In other words, to control for all of these confounding factors, it is enough to combine the before/after and the discontinuous policy variation so as to implement a difference-in-discontinuities design (see Grembi et al. 2016; Eggers et al. 2018).

Formally, consider a simple setup where the observed (budget) outcome is equal to the potential (budget) outcome associated with the set of treatments actually received by municipality *i* at time *t*, $Y_{it}=Y(\mathbf {T}_{it})$, where ${\mathbf{T}}_{{it}} \in {\text{IR}}^{{\text{3}}}$ is a 3-dimensional vector containing the realizations of three separate treatments: (1) the transfer cuts, that is, our treatment of interest; (2) the mayor’s wage; (3) the exemption from the DSP. This means that $\mathbf {T}_{it}$ can be decomposed as $\mathbf {T}_{it}=(C_{it},\mathbf {V}_{it}')'$, where *C* is a dummy capturing the fact of being subject to transfer cuts or not, and $\mathbf {V}_{it}$ is a vector containing the other two (confounding) treatments. As discussed above, treatment assignment sharply changes in population size, $P_{it}$, at the cutoff $P_c=5000$. In particular, at time $t=t_1$, the transfer cuts *C* are in place for municipalities above $P_c$, but not for those below $P_c$. The same cutoff, however, triggers a change in the confounding treatments too. Formally:

$$\begin{aligned} \mathbf {T}_{it}= \left\{ \begin{array}{ll} \mathbf {T}_{1v} &{} \quad \text{ if } P_{it} \ge P_c, t=t_1\\ \mathbf {T}_{0\tilde{v}} &{} \quad \text{ if } P_{it} < P_c, t=t_1 \end{array}\right. \end{aligned}$$

where $\mathbf {T}_{1v}=(1,\mathbf {v}')'$ and $\mathbf {T}_{0\tilde{v}}=(0,(\mathbf {1}-\mathbf {v})')'$, with $\mathbf {v}$ just indicating a vector of two dummy realizations.

In this setting, a simple (cross-sectional) RDD estimator cannot identify any causal effect of the transfer cuts *C* alone. In fact:

$$\begin{aligned} \hat{\tau }_{_{RDD}} = E\left[ Y(\mathbf {T}_{1v})-Y(\mathbf {T}_{0v})| P_{it} = P_c, t=t_1 \right] + E\left[ Y(\mathbf {T}_{0v})-Y(\mathbf {T}_{0\tilde{v}})| P_{it} = P_c, t=t_1 \right] , \end{aligned}$$

where the first term is one of the (local) average treatment effects of transfer cuts *C* that we may want to estimate, and the second is the bias introduced by the mayor’s wage and the DSP exemption.

To remove this bias and isolate the causal effect of *C* alone, we can exploit the time variation discussed above, that is, the fact that transfer cuts were implemented at $t_1=2012$ as they were not in place at $t_0<t_1$, while the other confounding policies are time-invariant in our sample. Formally:

$$\begin{aligned} \mathbf {T}_{it}= \left\{ \begin{array}{ll} \mathbf {T}_{1v} &{} \quad \text{ if } P_{it} \ge P_c, t=t_1\\ \mathbf {T}_{0v} &{} \quad \text{ if } P_{it} \ge P_c, t=t_0\\ \mathbf {T}_{0\tilde{v}} &{} \quad \text{ if } P_{it} < P_c\\ \end{array}\right. \end{aligned}$$

In addition to the standard continuity assumption of any RDD design, identification rests on the following assumption of *local parallel trends* (see Eggers et al. 2018):

$$\begin{aligned} \begin{array}{l} E\left[ Y(\mathbf {T}_{0v})-Y(\mathbf {T}_{0\tilde{v}})| P_{it} = P_c, t=t_1 \right] =E\left[ Y(\mathbf {T}_{0v})-Y(\mathbf {T}_{0\tilde{v}})| P_{it} = P_c, t=t_0 \right] ,\\ E\left[ Y(\mathbf {T}_{1v})-Y(\mathbf {T}_{1\tilde{v}})| P_{it} = P_c, t=t_1 \right] =E\left[ Y(\mathbf {T}_{1v})-Y(\mathbf {T}_{1\tilde{v}})| P_{it} = P_c, t=t_0 \right] . \\ \end{array} \end{aligned}$$

This assumption can be interpreted from two viewpoints. It states that the combined effect of the mayor’s wage and the DSP exemption ($\mathbf {v}$ vs $\mathbf {\tilde{v}}$), holding fixed the transfer cuts *C*, is time invariant. In other words, municipalities just above and just below $P_c$ would have been on parallel trends between $t_0$ and $t_1$ had transfer cuts *C* not been introduced at $t_1$. (Note that this assumption is more local than the standard parallel trends assumption of the difference-in-differences design, as it must hold only in the neighborhood of the policy threshold $P_c$.) From a different angle, the assumption states that the (time) difference in potential outcomes between $t_0$ and $t_1$, again holding fixed the transfer cuts *C*, must be continuous in population size at $P_c$. From this second perspective, the assumption is analogous to the RDD assumption of continuity in potential outcomes around the threshold.

Eggers et al. (2018) show that the above assumption is sufficient for identification. Under continuity and local parallel trends, the following difference-in-discontinuities estimator yields the (local) average treatment effect of policy *C* conditional on $\mathbf {V}=\mathbf {v}$:

$$\begin{aligned} \hat{\tau }_{_{DDISC}}\equiv & {} \big (\lim _{p \rightarrow P_c^+} E\left[ Y_{it} | P_{it} = p, t=t_1 \right] -\lim _{p \rightarrow P_c^-} E\left[ Y_{it} | P_{it} = p, t=t_1 \right] \big )\\&-\big (\lim _{p \rightarrow P_c^+} E\left[ Y_{it} | P_{it} = p, t=t_0 \right] -\lim _{p \rightarrow P_c^-} E\left[ Y_{it} | P_{it} = p, t=t_0 \right] \big ) \\= & {} E\left[ Y(\mathbf {T}_{1v})-Y(\mathbf {T}_{0\tilde{v}})| P_{it} = P_c, t=t_1 \right] -E\left[ Y(\mathbf {T}_{0v})-Y(\mathbf {T}_{0\tilde{v}})| P_{it} = P_c, t=t_0 \right] \\= & {} E\left[ Y(\mathbf {T}_{1v})-Y(\mathbf {T}_{0v})| P_{it} = P_c \right] . \end{aligned}$$

Note that the estimand identified above is conditional on specific realizations of the mayor’s wage and the DSP policy (i.e., $\mathbf {V}=\mathbf {v}$). In other words, the causal effect of transfer cuts on fiscal policy that we can identify with the estimator $\hat{\tau }_{_{DDISC}}$ refers to municipalities that are subject to the DSP fiscal rules and where mayors are paid a higher wage.

To implement the above difference-in-discontinuities estimator, we run two models: a local linear regression (LLR) with optimal bandwidth (see Calonico et al. 2014) and a 2nd-order local (spline) polynomial regression (LPR). In the case of LLR, given the optimal bandwidth $\Delta$, we restrict the sample to cities in the interval $P_i\in [P_c-\Delta ,P_c+\Delta ]$ and run the following model:

12 $$\begin{aligned} Y_{it}=\delta _0 + \delta _1\tilde{P}_{it} + C_i(\gamma _0+\gamma _1\tilde{P}_{it}) + A_t[\alpha _0+\alpha _1\tilde{P}_{it}+C_i(\beta _0+\beta _1\tilde{P}_{it})] +\varepsilon _{it} \end{aligned}$$

where $C_i$ is the treatment, $A_t=1$ after 2011, $\tilde{P}_{it}$ is the normalized population, and standard errors are clustered at the municipal level. The parameter $\beta _0$ identifies the after treatment effect.

In the case of LPR, we consider a more flexible functional form to fit the relationship between *Y* and *P* on either side of $P_c$ in the entire sample and run the following model:

13 $$\begin{aligned} Y_{it}=\sum _{k=0}^{p}(\delta _k \tilde{P}_{it}^k) + C_i\sum _{k=0}^{p}(\gamma _k \tilde{P}_{it}^k) + A_t\left[ \sum _{k=0}^{p}(\alpha _k \tilde{P}_{it}^k) + C_i\sum _{k=0}^{p}(\beta _k \tilde{P}_{it}^k)\right] +\varepsilon _{it} \end{aligned}$$

where again standard errors are clustered at the municipal level. Also in this case the parameter $\beta _0$ identifies the after treatment effect.

**Fig. 10:**
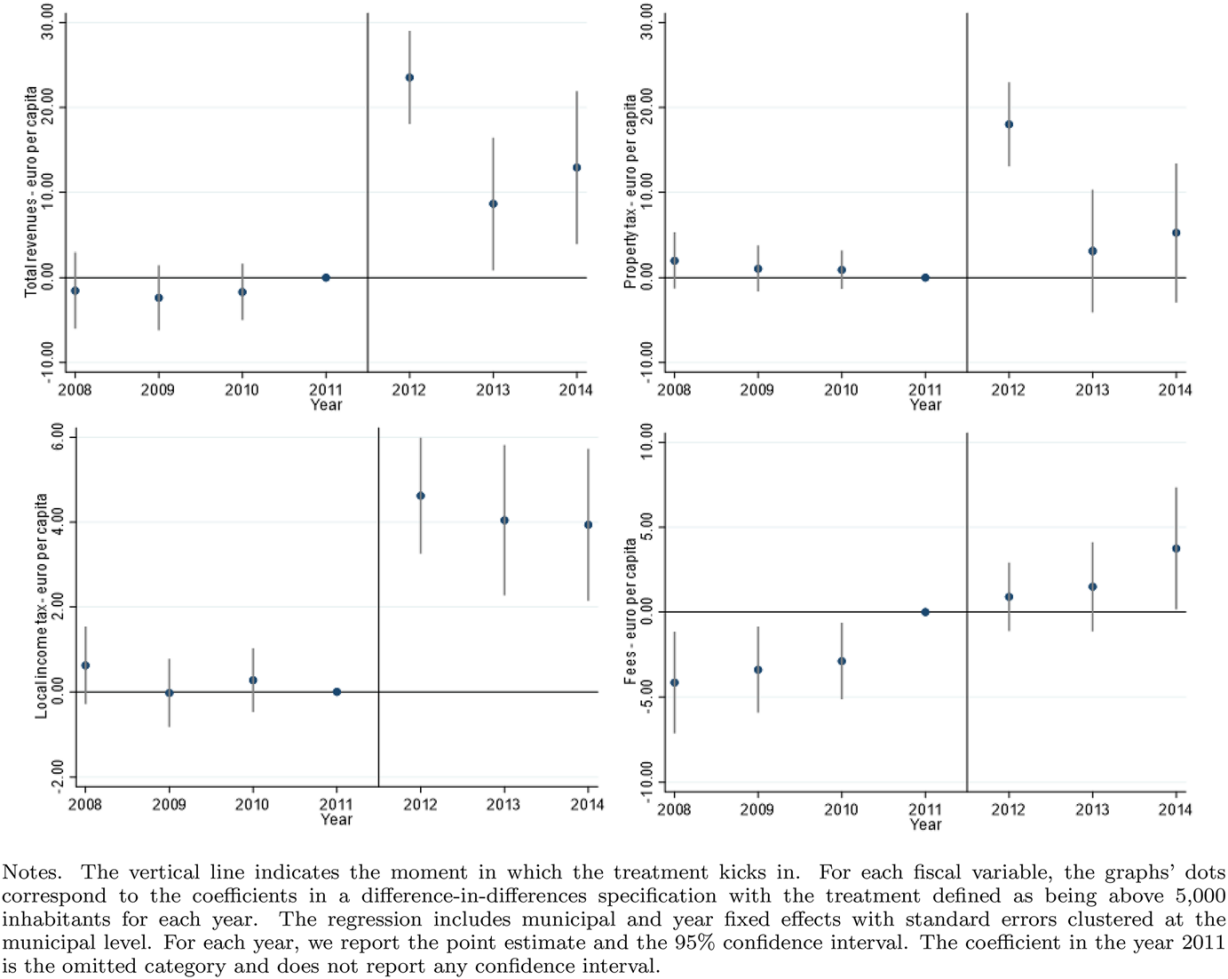
Tax revenues variables, pre and post-treatment trends

**Fig. 11:**
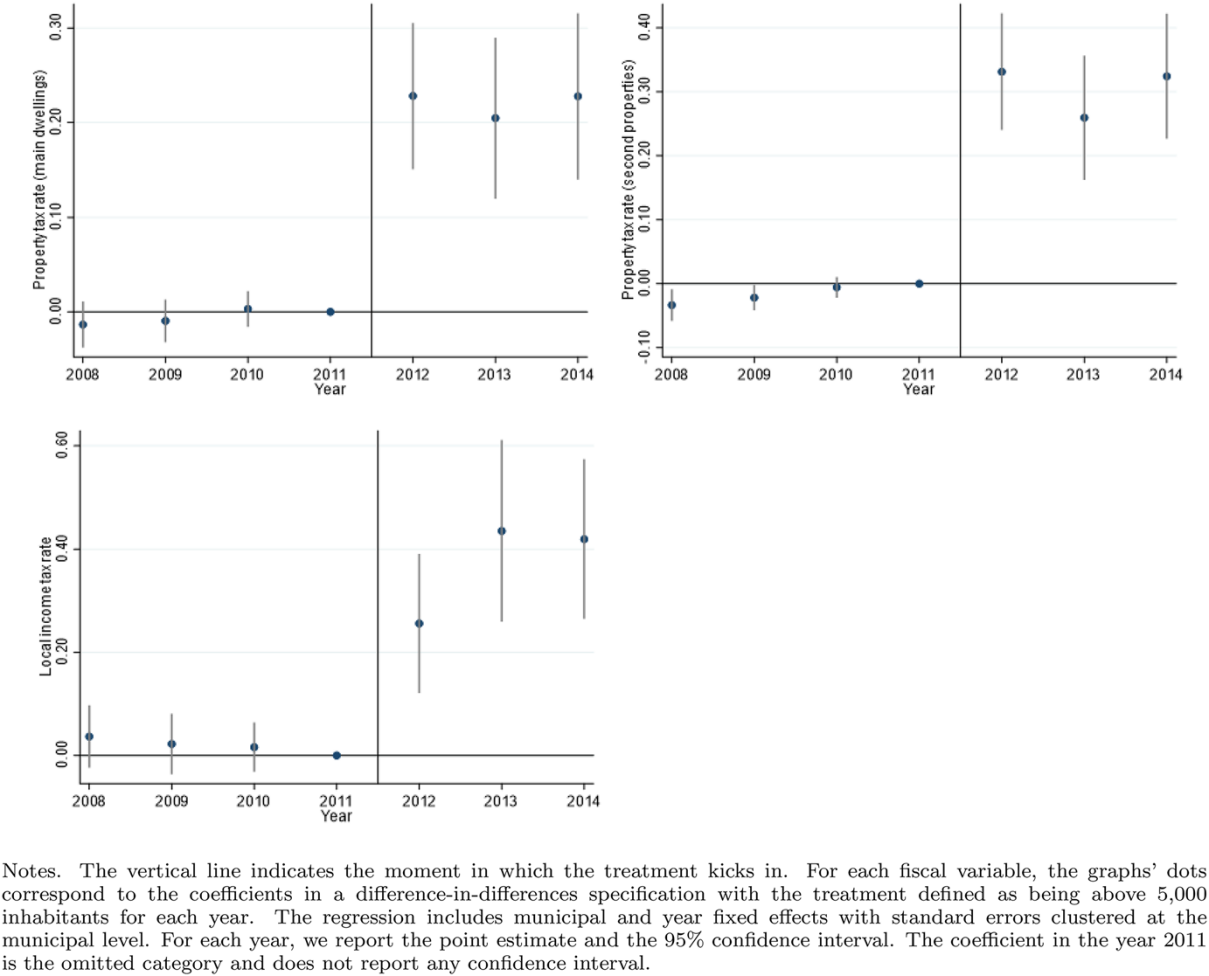
Tax rates variables, pre and post-treatment trends

## Empirical results

### Main findings

Tables 4 and 5 report the main results on the causal effect of transfer cuts on municipal governments’ composition of the fiscal adjustment. As a benchmark, we always report a standard difference-in-differences estimation (DID) in our restricted sample of all municipalities between 1000 and 10,000 inhabitants. We then provide the difference-in-discontinuities estimations with two different approaches: first, local linear regression (LLR), considering for each outcome variable the optimal population bandwidth around the 5000 inhabitants threshold following the algorithm developed by Calonico et al. (2014); second, with a local polynomial regression (LPR) considering a 1000 inhabitants bandwidth around the threshold^18^ and a second order polynomial approximation. Taking advantage of our dataset’s panel structure, we use the Within-the-Group estimator controlling for the municipal and year fixed effects and clustering standard errors at the municipal level. Finally, we provide results without and with control variables.

The point estimates show that municipal governments reacted to transfer cuts by increasing local tax revenues, rather than cutting expenditure. The size of this revenue based fiscal adjustment is larger in the neighborhood of the policy threshold, where we can identify a causal effect between 40 and 60 euros on per-capita tax revenues, depending upon the specification of the empirical model. As for the size, this tax increase ranges between 60 and 90% of total transfer cuts in 2012, between 8 and 12% of the average current expenditure, and between 15 and 22% of the average total tax revenues (measured for the treatment group of municipalities above 5000).

The point estimates are stable across all difference-in-discontinuities estimation methods, supporting the robustness of the findings. The increase in tax revenues is mainly due to an increase in revenues from the property tax (between 27 and 32 euros per capita), followed by a smaller increase in municipal fees (between 14 and 18 euros per capita) and municipal local income tax (between 8 and 11 euros per capita). In other words, we disclose a pass-through mechanism by which local governments react to the contraction of intergovernmental grants by increasing local taxes. As the fiscal policy variables we evaluate capture the sudden response of local governments to the fiscal shock, we identify a partial-equilibrium effect, which speaks directly to the theoretical hypotheses of the macro literature on fiscal consolidations. In other words, we can rule out general-equilibrium feedbacks that would make our results more difficult to interpret.^19^

Figure 5 reports, for the main financial variables, the graphical analysis of the discontinuities around the threshold of 5000 inhabitants, that is, the jump (if any) in the spline polynomial approximation. As expected, this analysis confirms the zero effect on local spending and the positive jump in tax revenues. Figure 6 provides the same graphical analysis of the discontinuities around the threshold of 5000 inhabitants for the main components of total tax revenues. All revenue components display a positive discontinuity, which—as expected—is more pronounced for the revenues from the property tax.

To study the potential redistribution implications of the higher tax burden, we investigate the magnitude of the treatment effect on four additional outcome variables: the statutory municipal income tax rate; a dummy that takes value one when we observe a progressive structure for the municipal income tax; and finally the two statutory tax rates of the property tax respectively on the residents main dwelling and the second properties owned, mainly, by non-residents. For each variable, we report the point estimates of DID, LLR and LPR estimators in Table 5 and the graphical analysis of the discontinuities around the threshold of 5000 in Fig. 7. The graphical analysis and the point estimates show a positive treatment effect (although the magnitude is comparable across model specifications, we lose the statistical significance in the LPR model). The highest increase (0.5 per thousand) is registered with the same magnitude in both types of property tax rates, suggesting the tendency to increase the tax burden equally on residents and non-residents. Although we observe a weaker treatment effect regarding the local income tax rate, there is positive (but not statistically significant) evidence in line with Alpino et al. (2020) that municipalities tend to increase the local income tax’s progressivity to cope with the transfers’ cuts.

As reported in Table 4 we observe a negative treatment effect on current expenditure statistically significant only in DID specification; this evidence corroborates the conclusion that the fiscal adjustment mainly follows the revenue channel. However, given the higher rigidity of expenditure, we decided to investigate also the possibility of a delay in the expenditure reaction considering as an additional specification a model where we consider as a dependent variable the current expenditure registered in each of the four years after 2012. The point estimates of this additional exercise, reported in Table 6, do not show any robust evidence of a delayed negative treatment effect on current expenditure.

**Table 7:** Heterogeneous effects by pre-treatment financial features, main outcome variables

		High fiscal effort			High Fiscal Capacity		
	Baseline	High	Low	Interaction	High	Low	Interaction
Local income tax	10.91*	$-$15.93**	15.26*	$-$31.19**	6.19	7.898	$-$1.708
Fees	17.87**	$-$2.94	27.35***	$-$30.29	66.312	8.082	58.23
Property tax	32.81*	$-$11.4*	51.91***	$-$63.31*	96.15*	25.29*	70.86*
Total tax revenue	61.60***	$-$30.27***	94.53***	$-$124.8***	168.67**	41.27**	127.4**
Capital expenditure	39.11	$-$127.2	41.30	$-$168.5	119	$-$21.70	140.7
Current expenditure	$-$17.45	$-$24.62	0.454	$-$25.08	45.31	$-$28.13	73.44

Municipalities between 1000 and 10,000 inhabitants; budget years between 2009 and 2012. The total number of observations corresponds to 13,795.

Heterogeneous difference-in-discontinuity estimates of the impact of grants reductions on fiscal variables in municipalities above 5000 inhabitants after 2011.

Estimation methods: 2nd-order spline polynomial approximation (LPR), as in Eq. (13) with a 1000 inhabitants bandwidth.

All policy outcomes are in euros per capita. Year and municipal fixed effects and control variables are included in all specifications.

Robust standard errors clustered at the municipality level are in parentheses. Significance at the 10% level is represented.

by *, at the 5% level by **, and at the 1% level by ***

### Heterogeneity analysis

To shed more light on the mechanism of the transmission from transfer cuts to higher local taxes, we go back to the implications of our theoretical framework and investigate if they are met in our data. Specifically, we perform heterogeneity analysis with respect to both pre-treatment fiscal variables and political variables. The empirical strategy is to interact the diff-in-disc specifications reported in Eqs. (12) and (13) with five dummies that identify the following municipal features measured in the pre-treatment period: (1) high fiscal effort in terms of legal tax rates; (2) high tax base (high capital endowment); (3) high political competition in terms of the incumbent margin of victory; (4) high party fragmentation in the mayoral coalition; (5) binding term limit for the incumbent. Each interaction is estimated with a separate regression. Although we are interested in analyzing the revenues side, each heterogeneity exercise is tested for all the outcome variables. The point estimate of the interacted treatment effect (that we can also define as the estimated heterogeneous treatment effect) is obtained using the LLR and the LPR estimators with standard errors clustered at the municipal level. All specifications include municipal fixed effects, time fixed effects and the complete set of controls. Since the LLR and LPR estimators provide similar results, we focus the rest of the analysis on the LPR specification results for simplicity.

Table 7 reports the heterogeneous treatment effects of the reduction in intergovernmental grants estimated considering municipalities with:

- *high vs low fiscal effort*, namely those local authorities that set the property tax rate or the local income tax rate above the 75th percentile of the distribution of the tax rates computed separately for each year and for each group of municipalities belonging to the same population bracket^20^. We considered the weighted average of the property tax and the municipal income tax rates, using as weights the national incidence of the two sources of revenues;
- *high vs low tax base*, namely those local authorities with a fiscal capacity above the 75th percentile of the distribution computed separately for each year and each municipality group belonging to the same population bracket. The fiscal capacity is the best proxy for the comprehensive tax base of each municipality. Our analysis is based on the Italian Ministry of Finance’s official values for the pre-treatment period considering the tax bases’ standard values and the standard tax rates.

In Table 7, for each financial outcome variable, we report three point estimates: (1) the treatment effects when the interaction dummy is zero identifying the treatment effect on municipality with low fiscal effort and low fiscal capacity (below the 75th percentile); (2) the interaction term between the treatment and the heterogeneous dummy; (3) finally, the sum of the two previous effects that identify the heterogeneous treatment effect on municipalities with high fiscal effort and high fiscal capacity (above the 75th percentile). The results reported in Table 7 show that, in line with our theoretical framework’s predictions, municipalities with low fiscal effort and high fiscal capacity are more prone to increase local taxes in reaction to the contraction of intergovernmental grants.^21^ In particular, municipalities with low fiscal effort exhibit a treatment effect on total tax revenues 50% higher than the entire sample (we observe a similar difference for the property tax, income tax and municipal fees). Instead, municipalities with low fiscal capacity show a treatment effect on total fiscal revenues 30% below the increase registered in the whole sample, a difference driven mainly by the property tax. Moreover, although point estimates of the treatment effect are not statistically different from zero, we can also observe that municipalities with high fiscal effort tend to reduce current and capital expenditure to offset cuts in grant.

Table 8 turns to the political expectations of our theoretical framework, and reports the heterogeneous treatment effects of the reduction in intergovernmental grants estimated considering municipalities with:

- *High vs low political competition*, measured by the margin of victory of the elected mayor;
- *Political fragmentation*, measured by the fact that the mayoral coalition is formed by a single party or by more parties (municipalities with high fragmentation are identified among those with a number of parties forming the coalition above the median of two parties);
- *Binding term limit for the incumbent*, when the mayor is in her or his second mandate (third mandate for municipalities below 3000 inhabitants) and can not run again in the next electoral competition.

The results reported in Table 8 show that political environments drive revenue-based fiscal consolidation. We observe that mayors tend to increase local taxes in reaction to transfer cuts when they face low political competition, no party fragmentation in the government coalition^22^, and a binding term limit (in this last case, however, we observe low statistical significance in the interaction term’s point estimates). Political concerns, hence, operate as a limit toward the increase of local taxes and especially the property tax given its extreme electoral salience^23^. In particular, point estimates show that the treatment effect on total tax revenues is 30% lower when the incumbent mayor faces high political competition and 60% lower in case of a fragmented majority. Instead, when the incumbent mayor can run for another mandate, the treatment effect on total tax revenues is 15% smaller. These results are in line with our theoretical prior. As tax hikes are faster to adopt and bring revenues more rapidly than expenditure cuts, a mayor may be prone to adopt them to realize the fiscal adjustment, unless she faces (external or internal) political competition by parties that have an incentive to emphasize the tax increase in the public discussion and campaign against the mayor, or she faces more stringent electoral concerns due to the possibility to be in office for another mandate.^24^

**Table 8:** Heterogeneous effects by pre-treatment political features, main outcome variables

		Political competition			Party fragmentation			Term-limit		
	Baseline	High	Low	Interaction	Yes	No	Interaction	Yes	No	Interaction
Local income tax	10.91*	14.424	10.58*	3.844	8.825***	12.97*	−4.145***	44.643	6.553	38.09
Fees	17.87**	−26.52	18.00**	−44.52	6.79	18.83**	−12.04	−12.39	12.57*	−24.96
Property tax	32.81*	17.04***	32.97**	−15.93**	−7.05***	33.67*	−40.72***	95.37	33.50**	61.87
Total tax revenue	61.60***	41.55***	61.55***	−20.00***	22.26***	65.47***	−43.21***	127.63	52.63***	75.00
Capital expenditure	39.11	65.15	14.90	50.25	−9.012	4.708	−13.72	−48.02	−71.48	23.46
Current expenditure	−17.45	−52.46***	−24.33	−28.13***	−33.041	−27.95	−5.091	−94.98	−16.82	−78.16

Municipalities between 1000 and 10,000 inhabitants; budget years between 2009 and 2012. The total number of observations corresponds to 13,795.

Heterogeneous difference-in-discontinuity estimates of the impact of grants reductions on fiscal variables in municipalities above 5000 inhabitants after 2011.

Estimation methods: 2nd-order spline polynomial approximation (LPR), as in Eq. (13) with a 1000 inhabitants bandwidth.

All policy outcomes are in euros per capita. Year and municipal fixed effects and control variables are included in all specifications.

Robust standard errors clustered at the municipality level are in parentheses. Significance at the 10% level is represented by *, at the 5% level by **, and at the 1% level by ***

### Robustness and validity checks

In this section, we evaluate the robustness of our main findings. First, we perform a set of permutation-based placebo tests to evaluate the possibility that our results arise from random chance rather than a causal relationship. Figure 8 reports the empirical c.d.f. of the point estimates from a set of difference-in-discontinuities estimations at 1000 false thresholds below and above the true threshold at 000 (namely, any point from 4900 to 3900 and any point from 5100 to 6100). The estimation method adopted for the placebo tests is the spline polynomial approximation with 2rd-order polynomial; the vertical lines indicate our benchmark estimate and its negative value as reported in Table 4. Focusing on the case of total revenues, where the possibility of false positive estimates is what worries us, the placebo exercise reported in the right-hand side panel of Fig. 8 shows that only 2.57% of the placebo estimates are above the baseline (true) estimate of 60 euros, thereby supporting the robustness of our results.

As discussed by Eggers et al. (2018), the difference-in-discontinuities design crucially rests on the assumption of local parallel trends between treatment and control units of observation in the neighborhood of the discontinuity. To address this issue, for each outcome variable, we estimate the difference between the treatment and the control groups for each year in our analysis plus two additional periods after 2012. This analysis does not impose the same 2012 data restriction as done in the diff-in-disc specification since the 2013 change in DSP rules is less relevant. In particular, we estimate the following model:

14 $$\begin{aligned} Y_{it}=\beta _0 + D_i[\beta _1+\sum _{t=2008}^{2014}(\beta _{2t} \eta _t)] + \sum _{t=2008}^{2014}(\beta _{3t}\eta _t) + \epsilon _{it} \end{aligned}$$

where $D_i$ is the treatment dummy, $\eta _t$ are the year dummies with year 2011 omitted to avoid multicollinearity. The model in (14) has been estimated using the *Within-the-Group* estimator controlling for the municipal fixed effect clustering the standard errors at the municipal level. Figures 9, 10 and 11 report the point estimates for the parameters $\beta _{2t}$ and the 95% confidence respectively for the expenditure variables, the revenues variables, and the tax rates variables. Evidence supporting the parallel trend assumption requires that we do not reject the $H_0: \hat{\beta _{2t}} = 0 \ \forall \ t = 2008, ...., 2011$ or, in other words, that the estimated distance of the outcome variables between the treatment and control group remains constant in the pre-treatment periods. This evidence is verified for all outcome variables except for capital expenditure in 2010 and municipal fees. However, capital expenditure is not in the analysis’s main focus, and municipal fees only marginally affect the test’s validity for total revenues.

**Fig. 12:**
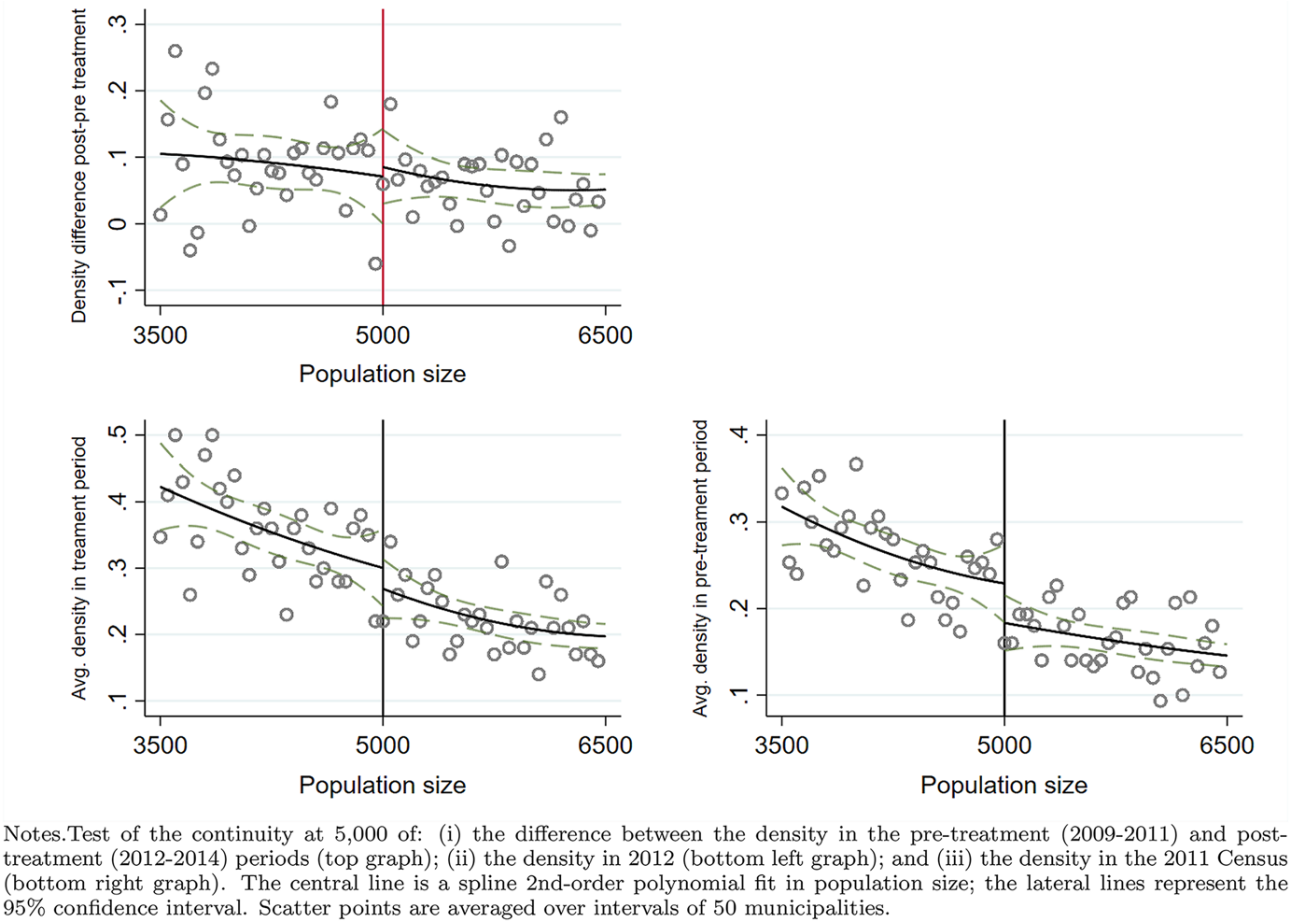
Density test of the discontinuity in the population threshold

In Fig. 12, in the spirit of McCrary (2008), we test the null hypothesis of continuity of the difference in the population density at 5000 between the pre-treatment (2009–2011) and post-treatment (2012–2014) periods (top graph) by drawing both scatters and (2nd-order) polynomial fits in population size. If mayors could manipulate population size and sort below the threshold to avoid the transfer cuts, our estimates would suffer from selection bias. However, this is not the case since there is no evidence of discontinuity in the density test (the point estimate of the discontinuity in the density test is 0.021 with a robust standard error of 0.081). Moreover, the manipulation would not have affected the outcome since the Ministry of Interior fixed the threshold according to the past census values that, by definition, could not be changed in this specific case. In Fig. 12, as further evidence, we also report the cross-sectional density tests for 2011 (bottom left) and 2012 (bottom right). Also, there, there is no evidence of manipulation.

**Fig. 13:**
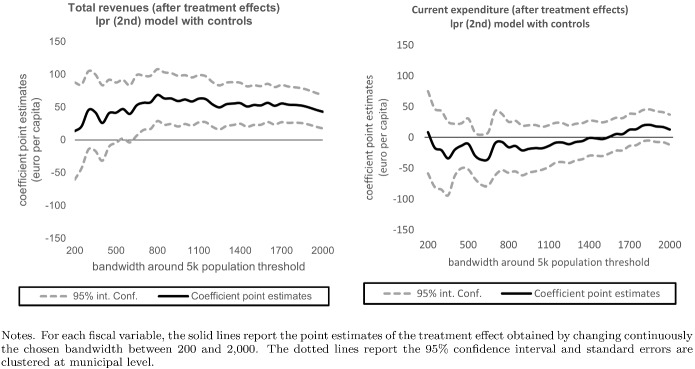
Sensitivity to continuous bandwidth increases between 200 and 2000

Figure 13, Tables 9 and 10 evaluate the sensitivity of the difference-in-discontinuities estimates to the bandwidth selection. Results are robust to the choice of different bandwidths. In particular, for the two main variable of interests (i.e., total tax revenues and current expenditure), Fig. 13 reports the point estimates obtained by changing continuously the chosen bandwidth between 200 and 2000. As far as the total tax revenues is concerned, the estimates are always above zero and fluctuate very little around the average of 50 euros, confirming the robustness of our main result. Instead, and again confirming the main finding, the point estimates on total current expenditure are never statistically different from zero.

Finally, Table 11 reports difference-in-discontinuities estimates for the main control variables, in order to test the absence of discontinuity in the main municipal structural features. In particular, the following variables are included in the test: the cadastral values, the percentage of main dwellings, the fiscal capacity of the property tax and the municipal income tax, the structure of population by age, the percentage of the mountain surface, the degree of urbanization, and a set of dummies to identify the geographical location of each municipality across the peninsula. As reported in the table, none of these variables show a non-zero discontinuity around the 5000 population threshold before and after the 2012 transfer cuts except cadastral values, fiscal capacity and population over 65 only in the DID specification.

**Table 9:** Sensitivity of all estimates to the bandwidth selection, main outcome variables

	LLR	LLR controls	LLR	LLR controls
	500 bwt	500 bwt	1000 bwt	1000 bwt
Local income tax	4.290	5.338	7.681*	7.893**
Fees	12.03	13.30	10.69*	10.70*
Property tax	34.28*	37.01**	22.51*	28.86***
Total tax rev.	50.60**	55.64***	40.88***	47.45***
Capital expend.	−3.233	22.89	19.13	25.78
Current expend.	−19.65	-22.28	10.19	11.13

Municipalities between 1000 and 10,000 inhabitants; budget years between 2009 and 2012. The total number of

observations corresponds to 13,795.

Difference-in-discontinuity estimates of the impact of grants reductions on fiscal variables in municipalities above 5000.

inhabitants after 2011 with different bandwidths.

Estimation methods: Local Linear Regression (LLR) as in Eq. (12).

All policy outcomes are in euros per capita. Year and municipal fixed effects are included in all specifications.

Robust standard errors clustered at the municipality level are in parentheses. Significance at the 10% level is represented.

by *, at the 5% level by **, and at the 1% level by ***

**Table 10:** Sensitivity of all estimates to the bandwidth selection, additional outcome variables

	LLR	LLR controls	LLR	LLR controls
	500 bwt	500 bwt	1000 bwt	1000 bwt
Local income tax rate	0.120*	0.122*	0.0810*	0.0724
Progressive income tax	$-$0.0789	$-$0.0710	$-$0.0275	$-$0.0185
Property tax rate (main residents)	0.646**	0.750***	0.459**	0.503***
Property tax rate (second properties)	0.495	0.517	0.279	0.288

Municipalities between 1000 and 10,000 inhabitants; budget years between 2009 and 2012. The total number of

observations corresponds to 13,795.

Difference-in-discontinuity estimates of the impact of grants reductions on fiscal variables in municipalities above 5000.

inhabitants after 2011 with different bandwidths.

Estimation methods: Local Linear Regression (LLR) as in Eq. (12).

All policy outcomes are in euros per capita. Year and municipal fixed effects are included in all specifications.

Robust standard errors clustered at the municipality level are in parentheses. Significance at the 10% level is represented.

by *, at the 5% level by **, and at the 1% level by ***

**Table 11:** Balance tests of control variables

	DID	LLR	LPR)
			(2nd order)
Cadastral values	−13.55***	12.43	22.2
*bandwidth*	All sample	980	1000
Percentage of main dwellings	0.0480	−0.163	−0.299
*bandwidth*	All sample	1003	1000
Fiscal capacity (property tax)	−0.658**	0.827	−0.122
*bandwidth*	All sample	1338	1000
Fiscal capacity (income tax)	−0.0784**	−0.00426	−0.0495
*bandwidth*	All sample	1022	1000
Population age 0-14	−0.0185	−0.131	−0.0942
*bandwidth*	All sample	1232	1000
Population age over 65	0.124***	0.0195	−0.112
*bandwidth*	All sample	1103	1000
Mountain surface (%)	0.141	6.522	13.78
*bandwidth*	All sample	880	1000
Degree of urbanization	0.00249	−0.0694	−0.223
*bandwidth*	All sample	956	1000
North west	0.00346	−0.0295	0.00757
*bandwidth*	All sample	727	1000
North est	0.00891	−0.0261	−0.0865
*bandwidth*	All sample	1513	1000
Center	−0.0057	0.0303	0.0183
*bandwidth*	All sample	1266	1000
South	−0.00338	0.0580	0.0980
*bandwidth*	All sample	1258	1000
Main islands	−0.00329	−0.0398	−0.0374
*bandwidth*	All sample	762	1000

Municipalities between 1000 and 10,000 inhabitants; budget years between 2009 and 2012. The total.

number of observations corresponds to 13,795.

Difference-in-discontinuity estimates of the impact of grants reductions on pre-treatment covariates in

municipalities above 5000 inhabitants after 2011.

Estimation methods: Local Linear Regression (LLR) with optimal bandwidth, as in Eq. (12);

2nd-order spline polynomial approximation (LPR), as in Eq. (13).

All policy outcomes are in euros per capita. Year and municipal fixed effects are included in all

specifications. Robust standard errors clustered at the municipality level are in parentheses.

Significance at the 10% level is represented by *, at the 5% level by **, and at the 1% level by ***

## Conclusion

Our results show that Italian municipalities reacted to the contraction of intergovernmental grants by mainly increasing local taxes rather than reducing expenditure. Thanks to the peculiarity of Italian institutions, we can claim that this effect is causal (internal validity) and can be extended to many other government settings with a certain degree of decentralization (external validity). The implications of the evidence we provide on this transmission mechanism are twofold. First, the macro literature on the output effect of fiscal consolidations should be extended to the analysis of local public finance. Second, the emergence of revenue vs expenditure based fiscal consolidations crucially rests on the forces at play in the underlying political environment, as we also find that the increase in local taxation is mainly driven by local governments with low electoral competition and low party fragmentation. Macro predictions on the policy responses to fiscal shocks should therefore incorporate both institutional and political factors.

Our results, moreover, can stimulate further research. In particular, in future research, we can expand the empirical analysis to investigate the impact of different fiscal adjustment behaviours on efficiency. It is not clear whether the pass-through mechanism based on the increase of local revenues may bust or deteriorate efficiency in providing local services, compared to the alternative pass-through mechanism that may stimulate local spending cuts. Evidence of different impact on efficiency may provide, in the end, normative implications in support of one of the two pass-through mechanisms.

## Footnotes

1 Other relevant studies in the fiscal adjustment literature include Woodford (2011), Favero and Giavazzi (2012), Auerbach and Gorodnichenko (2013), Ardagna and Caselli (2014).

2 Benzarti et al. (2020) study the asymmetric responses in the field of tax incidence reporting evidence that prices respond significantly more strongly to increases than to decreases in value-added taxes.

3 Prominent examples include Corbi et al. (2019), Surico and Trezzi (2019), Martin and Philippon (2017), Mian and Sufi (2014), Nakamura and Steinsson (2014), Chodorow-Reich (2019), Autor et al. (2013). See Guren et al. (2020) for further discussion of this literature.

4 Grembi et al. (2016) exploits the discontinuity at 5000 to evaluate the effectiveness of fiscal rules; Coviello et al. (2018) to estimate the impact of fiscal rules on firms’ dynamics at the local level; Gagliarducci and Nannicini (2013) to estimate the effect of the mayors’ wage on both political selection and performance, finding that better paid mayors are more educated and perform better once in office.

5 The fact that other studies use the threshold of 5000 inhabitants in Italy to estimate different causal effects does not affect the validity of our design-based evaluation, because none of the other policy discontinuities changed in 2012, as discussed in the section on the institutional background. And this is why our difference-in-discontinuities design identifies the effect of transfer cuts on fiscal adjustment only.

6 Other attempts at disaggregating fiscal adjustments can be found in Mertens and Ravn (2013), Romer and Romer (2016), and Perotti (2014). These papers, however, are limited to the U.S. and often only consider either the tax or the spending side of fiscal corrections.

7 Brollo and Nannicini (2012) implements a close-race regression discontinuity design in Brazil and finds that municipalities in which the mayor is affiliated with the party of the Brazilian president receive larger discretionary transfers for infrastructures. Herwatz and Theilen (2014) focus on Germany and find some role for political affiliation of the state governor in determining the distribution of transfers. Bracco et al. (2015) find a similar result for Italy, where municipalities ruled by a mayor affiliated to one of the parties in power at the central level receive a larger amount of grants than non-aligned mayors. Vega and Vega (2013) focus on Portugal and finds that the distribution of transfers is affected by the political cycle.

8 Our paper also indirectly contributes to the literature on the effects of balanced budget rules, see—among others—Grembi et al. (2016), Azzimonti et al. (2016), Asatryan et al. (2018), Eliason and Lutz (2018).

9 As in Gong and Zou (2002) and Mieszkowski (1972) in our framework capital includes real estate property.

10 Formally, the representative agent maximizes its utility by taking as given governments’ fiscal choices; then the local government maximizes its own welfare function by incorporating the agent’s optimal condition; and finally the central government maximizes welfare by taking into account the other two players’ FOCs.

11 This result, which predicts a positive relationship between the (pre-existing) capital endowment and the property tax rate change is consistent with Kopczuk and Slemrod (2002) and Kopczuk (2005) who emphasize that the dead-weight loss of a tax rate increase is relatively lower when the tax base is higher.

12 The electoral system is different according to the population: in small municipalities (below 15,000 inhabitants) there is single-round plurality system; instead, in larger municipalities (above 15,000 inhabitants) there is a run-off system. See Galasso and Nannicini (2015) for further details on Italian politics.

13 For a detailed analysis on the impact of the DSP on local governments’ fiscal policy, see Grembi et al. (2016). On the impact of the DSP on the dynamics of firms’ strategies, see Coviello et al. (2018).

14 On the impact of financial crises on public policy, see Gokmen et al. (2018).

15 Total current expenditure does not include the outlays related to waste management and local public transports, these items have been subtracted because in a large number of municipalities they are externalized and are not reported in the municipal budget account.

16 Property tax rates are differentiated between main dwellings (owner-occupied main residence), where the legal tax rates could be set in a 0.2-0.6% range, and secondary properties where the legal tax rates could be set in a 0.46%-1.06% range. The tax base corresponds to the cadastral value of the properties without differentiation between main dwellings and secondary properties.

17 Municipal income tax could be set on the top of the national marginal tax rate between a 0-0.8% range, moreover the municipalities can also opt for a progressive increase.

18 In Sect. 5.3 we also discuss the sensitivity of the bandwidth selection as a robustness check.

19 See Guren et al. (2020) on how to disentangle partial and general equilibrium effects in cross-regional empirical estimates in macroeconomics.

20 The population brackets considered in the analysis are: between 1000 and 2000, between 2000 and 3000, between 3000 and 5000, and finally between 5000 and 10,000.

21 In line with our results Rubolino (2020) provides evidence that positive shocks on the tax base lead Italian municipalities to raise taxes on property and income.

22 On the impact of political fragmentation on fiscal adjustment, see Alesina et al. (1998), Perotti and Kontopoulos (2002), Schaltegger and Feld (2009), Grier et al. (2015), Artés and Jurado (2018).

23 On the electoral salience of the property tax, see Bracco et al. (2019)

24 We detect, instead, no effect of political ideology (left vs right) on the composition of fiscal adjustment (on the impact of ideology on fiscal adjustment, see Tavares (2004)). However, we believe that this last exercise’s power is probably hindered by the presence of local parties (*liste civiche*) that can not be linked to the two national coalitions in 73% of municipalities of our sample. Therefore, the political ideology is not reported in the paper and would be available only on request.
